# Physiological roles of lignins – tuning cell wall hygroscopy and biomechanics

**DOI:** 10.1111/nph.70505

**Published:** 2025-10-15

**Authors:** Edouard Pesquet, Igor Cesarino, Shinya Kajita, Katharina Pawlowski

**Affiliations:** ^1^ Arrhenius Laboratories, Department of Ecology, Environment and Plant Sciences (DEEP) Stockholm University Svante Arrhenius väg 20A 106 91 Stockholm Sweden; ^2^ Bolin Centre for Climate Research Stockholm University Svante Arrhenius väg 20A 106 91 Stockholm Sweden; ^3^ Stockholm University Center for Circular and Sustainable Systems (SUCCeSS) Stockholm University Svante Arrhenius väg 20A 106 91 Stockholm Sweden; ^4^ Department of Botany, Institute of Biosciences University of São Paulo Rua do Matão, 277 – CEP: 05508‐090, Butantã São Paulo Brazil; ^5^ Graduate School of Bio‐Applications and Systems Engineering Tokyo University of Agriculture and Technology Tokyo 184‐8588 Japan

**Keywords:** antioxidant, biomass recalcitrance, cell wall biomechanics, cell wall hygroscopy, chemical and structural control of lignin, impermeability, lignin topochemistry, spatio‐temporal regulation of lignins

## Abstract

Lignins constitute the second most abundant carbon‐storing biopolymers in the biosphere. These phenolic polymers accumulate in different concentrations, compositions, and localisations within and between cell wall layers and cell types. Lignins were acquired during plant terrestrialisation 450 million years ago, and the diversification of their chemistries and structures during plant evolution and speciation allowed plant cells to adjust and/or gain new functions for facing developmental and environmental challenges. The main property conferred by any lignin polymer is to modify the hygroscopic capacities of plant cell walls to set their responsiveness to changes in water content. To do so, lignin accumulation increases the impermeable, antioxidant, recalcitrant and/or mechanical properties of cell walls to modify their water responsiveness. Adjusting these diverse properties depends on the chemistry, structure and distribution pattern of the lignin polymers, collectively named topochemistry. Lignin topochemistries are differently regulated spatially and temporally for each cell type. In this review, we provide a unifying description of lignins as regulators of cell wall hygroscopy and biomechanics for plant physiology as well as describe the molecular and cellular processes, enabling each cell wall layer to specifically adjust lignin properties.

## Sammanfattning

Ligniner utgör de näst vanligaste kollagrande biopolymererna i biosfären. Dessa fenoliska polymerer ackumuleras i olika koncentrationer, sammansättningar och lokaliseringar inom och mellan cellväggsskikt och celltyper. Ligniner förvärvades under växternas terrestrialisering för 450 miljoner år sedan. Diversifieringen av deras kemi och strukturer under växternas evolution och artbildning gjorde det möjligt för växtceller att anpassa sig och/eller få nya funktioner för att möta utvecklings‐ och miljöutmaningar. Den huvudsakliga egenskapen som ges av en ligninpolymer är att modifiera de hygroskopiska kapaciteterna hos växtcellväggar för att justera deras förmåga att anpassa sig till förändringar i vattenhalten. För att göra detta ökar ligninackumulering de ogenomträngliga, antioxidanta, motsträviga och/eller mekaniska egenskaperna hos cellväggarna för att anpassa deras interaktiom med vatten. Justering av dessa olika egenskaper beror på ligninpolymerernas kemi, struktur och distributionsmönster, gemensamt kallat topokemi. Lignintopokemier regleras olika rumsligt och tidsmässigt för varje celltyp. I denna översikt ger vi en samlad beskrivning av ligniner som regulatorer av cellväggshygroskopi och biomekanik för växtfysiologi samt beskriver de molekylära och cellulära processer som gör det möjligt för varje cellväggslager att specifikt justera lignins egenskaper.

## Résumé

Les lignines sont des biopolymères phénoliques occupant la seconde place en terme d'abondance dans notre biosphère. Ces polymères diffèrent en concentration, composition et localisation au sein et entre les diverses couches pariétales et les divers types cellulaires. Les lignines, apparues il y a 450 millions d'années lors de la terrestrialisation des végétaux, se sont diversifiées chimiquement et structurellement au cours de l'évolution et de la spéciation, permettant aux cellules végétales d'acquérir de nouvelles fonctions pour faire face aux contraintes développementales et environnementales de la vie terrestre. La propriété principale des lignines est de contrôler les capacités hygroscopiques des parois afin d'ajuster leur réponse face aux changements hydriques. Ainsi, l'accumulation de lignines augmente l'imperméabilité des parois ainsi que leurs propriétés antioxydantes, leur récalcitrance et/ou leurs propriétés mécaniques tout en conférant une capacité de réponse face aux changements hydriques. L'ajustement de ces différentes propriétés dépend des modifications chimiques, structurelles et de la distribution spatiale des lignines – ceci est englobé dans le concept de topochimie. La topochimie des lignines est régulée tant spatialement que temporellement au niveau de chaque type cellulaire. Dans cette revue de synthèse, nous établissons une description unificatrice des lignines en tant que régulateurs des propriétés hygroscopiques et mécaniques pariétales nécessaires à la physiologie végétale. Nous décrivons également les processus moléculaires et cellulaires permettant à chaque couche pariétale d'ajuster les propriétés de leurs lignines.

## Resumo

As ligninas constituem o segundo biopolímero mais abundante na biosfera. Esses polímeros fenólicos se acumulam em diferentes concentrações, composições e localizações dentro e entre as camadas da parede celular e os diferentes tipos de células. As ligninas foram adquiridas durante a conquista do meio terrestre pelas plantas cerca de 450 milhões de anos atrás, e a diversificação de suas estruturas químicas durante a evolução permitiu que as células vegetais ajustassem e/ou ganhassem novas funções para enfrentar os novos desafios do meio terrestre. A principal característica conferida por qualquer polímero de lignina é modificar as propriedades higroscópicas das paredes celulares das plantas para definir sua capacidade de resposta às mudanças no teor de água. Para isso, o acúmulo de lignina aumenta as propriedades impermeáveis, antioxidantes, recalcitrantes e/ou mecânicas das paredes celulares. Ajustar essas diversas propriedades depende da composição química, da estrutura e do padrão de distribuição dos polímeros de lignina, o que é coletivamente chamado de ‘topoquímica’. A topoquímica das ligninas é regulada de forma diferente espacial e temporalmente para cada tipo de célula. Nesta revisão, fornecemos uma descrição unificadora das ligninas como reguladoras da higroscopia e da biomecânica da parede celular para a fisiologia das plantas, bem como descrevemos os processos moleculares e celulares que permitem que cada camada de parede celular ajuste especificamente as propriedades da lignina.

## Zusammenfassung

Lignine, die zweithäufigsten Kohlenstoff‐speichernden Biopolymere, akkumulieren in unterschiedlichen Konzentrationen, Zusammensetzungen und an unterschiedlichen Orten innerhalb und zwischen Zellwandschichten und Zelltypen. Lignine entstanden während der Evolution von Landpflanzen vor 450 Millionen Jahren, und die Diversifizierung ihrer Struktur und Zusammensetzung im Zuge der Evolution ermöglichte Landpflanzen die Adjustierung vorhandener bzw. den Erwerb neuer Funktionen für Entwicklung und Anpassung an Umweltbedingungen. Der Haupteffekt eines Lignins ist die Modifizierung der hygrosckopischen Eigenschaften der pflanzlichen Zellwand, die die Reaktion auf Änderungen im Wassergehalt bestimmen. Für diesen Zweck verstärkt die Ansammlung von Ligninen die antioxidativen und mechanischen Eigenschaften der Zellwände sowie ihre Permeabilität und verändert damit ihre Reaktion auf Wasser. Die Adjustierung dieser Eigenschaften beruht auf der chemischen Zusammensetzung, Strukut und Verteilung unterschiedlicher Lignine, d.h. auf ihrer Topochemie. Die Topochemie von Ligninen ist für jeden Zelltyp räumlich und zeitlich geregelt. In diesem Review erstellen wir eine vereinheitlichte Beschreibung von Ligninen als Regulatoren der Hygroskopie von Zellwänden und der physiologisch erforderlichen Biomechanik und beschreiben die molekulären und zellulären Prozesse, die es ermöglichen, die Eigenschaften on Lignin in jeder Zellwandschicht spezifisch zu adjustieren.

## まとめ

リグニンは、炭素の貯蔵に貢献する生物圏で2番目に豊富な生体高分子である。このフェノール性ポリマーは、植物の異なる種類の細胞や細胞壁層内において濃度や化学組成、局在性に違いがある。リグニンは450億年前に植物が陸地へ上がった際に獲得され、進化と種分化の過程でその化学的性質と構造が多様化したと考えられる。陸上で植物細胞が直面したであろう環境変化の変化に適応し、新たな機能を獲得することにリグニンは大いに役立ったはずである。リグニンポリマーが果たす主な役割は、植物細胞壁の吸湿能力を変化させ、含水量の変化に対する応答性を設定することである。そのために、リグニンの蓄積は、細胞壁の不透水性、抗酸化性、難透水性、機械的強度を増加させ、細胞壁の水分応答性の向上に貢献する。これらの多様な特性の調節は、リグニンポリマーの化学、構造、分布パターン(トポケミストリーと総称される)に依存する。リグニンのトポケミストリーは、細胞タイプごとに時空間的に異なる制御を受けている。本総説では、植物生理学における細胞壁の湿度調節とバイオメカニクスの調節因子としてのリグニンについて統一的な説明を行うとともに、各細胞壁層がリグニンの特性を特異的に調節することを可能にする分子および細胞プロセスについて言及する。

**Table:** 

	Contents	
	Summary	2674
I.	Introduction	2675
II.	Adjusting cell wall hygroscopy with lignins	2675
III.	The controlled combinatorial assembly of lignin polymers	2678
IV.	Topochemical control of lignin formation	2687
V.	Spatial and temporal distribution of lignins with specific topochemistries	2690
VI.	Tuning the hygroscopy of cell walls with lignins to adjust biomechanical properties	2693
VII.	Adjusting lignin topochemistry to diversify cell type and tissue functions	2695
VIII.	Conclusions and perspectives	2698
	Acknowledgements	2699
	References	2699

## I. Introduction

**Fig. 1:**
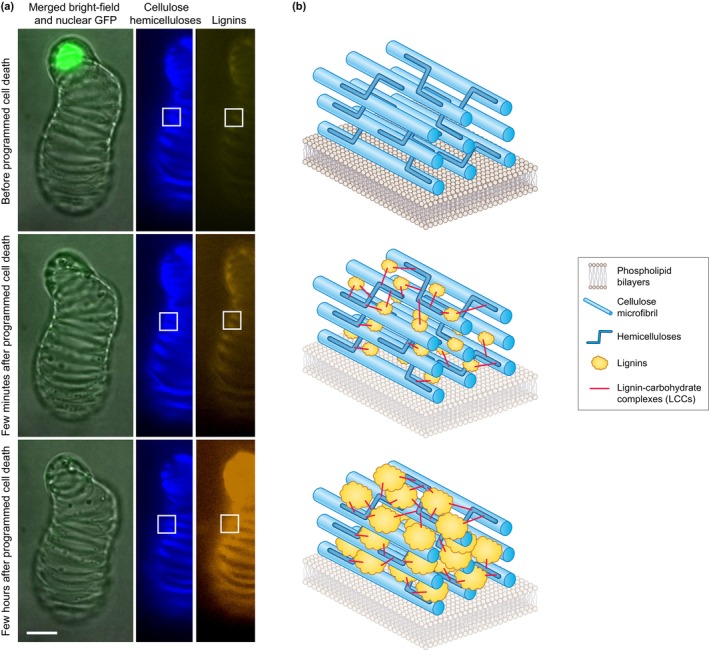
Lignin accumulation in cell walls differentially covers the surface of polysaccharides and fills in the cell wall void spaces to dynamically adjust cell wall hygroscopic and mechanical properties. (a) Real‐time live cell imaging showing the delayed lignin deposition onto the secondary cell wall polysaccharides was performed using inducible pluripotent cell suspension cultures of *Arabidopsis* triggered to differentiate into tracheary elements (Ménard *et al*., 2024). Bar, 10 μm. Note that programmed cell death is monitored by the presence of nuclear green fluorescent protein (GFP), and that cell wall polysaccharides (cellulose and hemicelluloses) labelled with fluorescent wheat germ agglutinin (WGA) are shown in blue, whereas lignins detected by their autofluorescence are shown in yellow/orange (Decou *et al*., 2017). Live imaging modified from Pesquet *et al*. (2010). (b) Scheme showing the gradual lignin accumulation initiating and progressing once cell wall polysaccharides have been deposited so that lignin impregnates the surface of other cell wall polymers and the void spaces in the cell walls. Although the exact nature of the lignin initiation sites is not known, previous observations on tracheary elements suggest the existence of multiple initiation sites (Nakashima *et al*., 1997) that are gradually extended to enable lignification to progress independently of cell viability (called *postmortem* lignification for xylem tracheary elements) (Pesquet *et al*., 2013). Lignin–carbohydrate complexes (LCCs) represent intermolecular covalent linkages are shown as red lines. Note that in the schematic representation, the phospholipid bilayer of the plasma membrane is shown in lighter colours in dead cells, but it would disappear completely after programmed cell death as previously shown by Ménard *et al*. (2015).

Lignins represent the second most abundant class of biopolymers on Earth (Boerjan *et al*., 2003). Lignins are phenolic polymers that accumulate in cell walls of specific cell types in some algal and all terrestrial and aquatic vascular plant species (Boerjan *et al*., 2003; Martone *et al*., 2009). They represent one of the youngest groups of biopolymers and were acquired by plants 450 million years ago during the Devonian period concomitantly with the evolutionary outburst of terrestrial vascular plants (Weng & Chapple, 2010; Blaschek *et al*., 2024). Lignins are hypothesised to derive either from melanin‐type polymers – oxidatively assembled polymers of phenol/catechol amino acids (Glagoleva *et al*., 2020; Blaschek & Pesquet, 2021) already present in all living species; and/or from amino acid esters of phenylpropanoid acids already present in algae and mosses (Labeeuw *et al*., 2015; Renault *et al*., 2017, 2019); and/or polymers of flavonoids already present in mosses (such as poly‐apigenin in *Hypnum cupressiforme*; Rencoret *et al*., 2021). Unlike the ubiquitous presence of polysaccharides in all cell wall layers of all algal and plant cells, lignins specifically accumulate in distinct cell wall layers of specific cell types to support the novel cellular functions required for terrestrial growth. The evolutionary novelty of lignins is unique among biopolymers as they are formed using enzymatic and/or nonenzymatic oxidations to assemble phenolic monomers into optically inactive and apparently nonorientated polymers whose structure (e.g. backbone length, branching degree and size) is not guided by any protein scaffold (Box 1). During cell wall formation, lignins are deposited after the structural polysaccharides, gradually from the oldest to the newest layers (Donaldson, 2001; Pesquet *et al*., 2010, 2013). Although still largely unknown in their chemical natures, initiation sites located in specific cell wall layers will allow lignin polymers to differently cover the surface of cell wall polysaccharides and partly fill in the void spaces in between these polysaccharides (Fig. 1). Alternatively, extending lignin polymers started without fixed initiation sites will anchor to specific cell wall layers, positioning some of their aromatic rings parallel to cellulose microfibrils (Atalla & Agarwal, 1985) and interacting covalently or not with other cell wall components (Fig. 1). The differential impregnation with lignins will control the hygroscopic capacity of cell walls by adjusting both the accessible water adsorption sites on polysaccharide surfaces and the capillary retention of water in void spaces. This will consequently contribute to adjusting the biochemical and biomechanical properties of specific cells and tissues to fulfil their physiological roles.

Box 1 **Glossary**

Lignins are the most abundant phenolic polymers in the biosphere, first among other phenolic biopolymers, such as tannins, melanins and suberins that are all present in plants. The key terminology regarding lignins includes the following:

*Lignin‐related genes/proteins:* these genes/proteins encompass those that are associated with all aspects of lignification, including (a) regulatory proteins that control both developmental and stress lignification; (b) biosynthetic genes involved in the production of lignin intermediates and precursors, as well as other related phenolic metabolites; (c) transporters, considering that at least part of the lignin precursors are transported to the cell wall via transmembrane transporters; and (d) phenoloxidases, which are associated with radical formation for lignin oxidative assembly. Most of these genes belong to multigenic families in which not all members are necessarily involved in lignification (e.g. phenoloxidases) or whose members might have diversified through subfunctionalisation (e.g. developmental *vs* stress lignification). DIRIGENT proteins (DIRs) may be classified in both categories (b) and (d).

*Lignification:* this term describes the complete process of lignin formation and assembly in cell walls that includes: the intracellular generation of lignin precursors and their transport to the cell wall; the extracellular activation of lignin precursors into radicals, and their combinatorial assembly into phenolic polymers and sequestration in specific cell wall layers. To date, it remains unknown how lignin polymers are gradually formed in cell walls as the identity of initiating lignin sites (for ‘primer/nuclei’ and/or ‘oligo/polymer anchoring’), the number of extending polymer ends, the number and size of lignin branches, the rate of lignin end‐wise extension and the degree of polymerisation of lignins are still unknown.

*Lignin monomers/precursors/(sub)units:* ‘Lignin monomers’ corresponds to the individual free diffusing components that will be oxidatively assembled during lignification into lignin polymers. The distinction between lignin monomers and precursors is still unclear, as some intermediates of the biosynthetic pathway can be incorporated either *in vitro*, *in vivo* or upon pathway perturbations; and providing phenoloxidases, even if not physiologically associated with lignification, with distinct phenolic compounds enables the *in vitro* formation of polymers that resemble lignin polymers. To date, the terms lignin monomers and lignin precursors are synonymous as the molecular form(s) in which building blocks are provided for lignification have not been physiologically identified. ‘Lignin (sub)units’ correspond to the incorporated forms of the monomers into the lignin polymer.

*Lignin unit nomenclature:* historically, lignins were believed to be made exclusively of monolignols (phenylpropane alcohols) due to their high abundance, which even sometimes led some authors to refer to lignins as polylignols. Analytical methods (such as pyrolysis, nitrobenzene oxidation or KMnO_4_ oxidation) were incapable of resolving the aliphatic group of each lignin unit but could only reliably distinguish the C_6_ aromatic groups. These destructive analyses generate different *ortho*‐substituted phenols, including *p*‐hydroxyphenyl (H), guaiacyl (G) and syringyl (S) groups, previously used to discriminate units (Boerjan *et al*., 2003). Improvements in the sensitivity of nondestructive analytical methods (e.g. Two‐Dimensional Heteronuclear Single Quantum Coherence Nuclear Magnetic Resonance, 2D NMR HSQC) revealed that lignins incorporate many noncanonical units that have similar C_6_ aromatic ring substitutions but differ in their aliphatic group size, proportion of unsaturation and function. Even with recent methods, linking aliphatic chain and function with the aromatic ring substitution for each unit is nevertheless difficult. Some authors have addressed these limitations by extending the nomenclature to G' or S′ referring sometimes to G or S units with α ketone or deriving from coniferaldehyde or sinapaldehyde monomers, X2 as phenylpropene units with terminal aldehyde, FA or *p*CA for G or H units with propene aliphatic chains with terminal carboxylate or ester functions (Table 1). To unify historical nomenclature with current knowledge and avoid increasing the complexity of the lignin letter coding, we merged previously used terminology with the nomenclature used for phenolic compounds called (neo)lignans (Moss, 2000), to group all lignin units by their C_6_ aromatic ring substitution levels, by the size of their aliphatic group C_X_, and by their terminal function (Table 1). This nomenclature system has the benefit of being inclusive for all natural and artificial lignin units, extendable without increasing the letter code complexity, agrees with the original biochemical terminology and enables an easy understanding of which unit is incorporated. To be adopted by all, our nomenclature would require a consensus among lignin scientists. Note that correspondences between monomer, units and previously used lignomics and HSQC terminology are provided in Table 1.

*Lignin topochemistry:* this term describes both the chemical features (type of chemical groups/functions, unsaturation, linkages and position for each unit) and the distribution of lignins in different layers of the cell wall (both primary and secondary) of different cell types.

*Condensed and uncondensed lignin interunit linkages:* historically defined by the number of free aromatic protons (Ludwig, 1971), units interlinked with C‐C bonds on C_6_ aromatic group other than the C1 position, such as β‐5/phenylcoumaran and 5‐5/biphenyl (thus only presenting two free aromatic protons) are called condensed lignin linkages (Ralph *et al*., 2019). By contrast, ‐C‐C‐ and ‐C‐O‐C‐ linkages between C_3_ aliphatic and/or the *para* position of C_6_ aromatic groups, such as the β‐*O*‐4/β‐aryl ether, 4‐*O*‐5/diphenyl ether, β‐β/resinol and β‐1/diaryl propane (thus presenting three free aromatic protons), are defined as uncondensed lignin linkages (Ralph *et al*., 2019). Note that generally condensed linkages are more difficult to break down chemically than uncondensed ones, except for 4‐*O*‐5/diphenyl ether. As α‐*O*‐4/α‐aryl ether linkages have three free aromatic protons and can be cleaved easier than β‐*O*‐4 linkages (Kim *et al*., 2011; Jasiukaitytė‐Grojzdek *et al*., 2020), these could also be grouped among the uncondensed linkages (Ralph *et al*., 2019).

*Mono‐/oligo‐/polylignols:* although monolignols define *sensu stricto* phenylpropenes with a terminal alcohol function, the term is sometimes wrongly used to encompass all phenylpropenes and even to regroup all lignin monomers. This wrongful definition leads some authors to assimilate lignin polymers as polylignols. However, this ‘lignol’ terminology cannot be used restrictively for lignins, as many *bona fide* di‐ to oligomeric compounds formed from monolignols such as (neo)lignans are neither lignins nor their biosynthetic intermediates (Moss, 2000).

*Industrial/technical lignins, synthetic lignins and native lignins:* whereas native lignins (also called protolignins in older articles) correspond to the lignins naturally assembled in plant cell walls, the term technical or industrial lignins describes phenolic polymers isolated from different biomass sources using mechanico‐thermochemical extraction methods. Synthetic lignins (also called dehydrogenation polymers or DHPs) derive from the *in vitro* oxidative polymerisation of lignin monomers using phenoloxidases or other oxidants. Industrial/technical lignins such as milled wood lignins, kraft lignins and lignosulfonates share some similarities with native lignins (such as C_6_ substitution levels) but greatly differ in molar weight, interunit linkage types and proportion as well as aliphatic functional groups. Synthetic lignins share more structural similarities with native lignins but differ in composition, molar weight and interunit linkage proportions. The biological relevance of using synthetic and/or technical lignins to understand native lignins is therefore limited as it cannot account for native lignin spatial differences at the subcellular and cellular levels.

## II. Adjusting cell wall hygroscopy with lignins

Cell wall hygroscopy is the property characterising the water attracting/releasing capacity of each cell wall layer for each cell type. It is an essential component of plant physiology contributing to the hydro‐mineral conduction of xylem sap (Ménard & Pesquet, 2015) and affecting the permeability of seed coats (Yonekura‐Sakakibara *et al*., 2021). Cell wall hygroscopic capacity is defined by the matric potential Ψ_m_ – a component of the water potential Ψ_w_ (Boyer, 1967; McClendon, 1981; Thompson, 2008) – that characterises the pressure required to displace the cell wall‐bound water to adjoining permeable sites (cytoplasm, vacuole, external environment). Water will thus be gained or released from cell walls depending on its Ψ_w_ differences with adjoining sites within and/or between cell types (Boyer, 1967; McClendon, 1981; Thompson, 2008). The tuneable properties conferred by lignins are key elements to quasi‐irreversibly set the hygroscopy of each cell wall layer in each cell type in response to developmental and/or environmental conditions (Ménard & Pesquet, 2015; Emonet & Hay, 2022). The adjustment of lignin‐dependent cell wall hygroscopy relies on the following:

1. The spatial distribution of lignins, which can be controlled within and between cell wall layers for each cell type. An example is in xylem sap conducting tracheary elements where pits with primary cell wall remain unlignified and interspersed among lignified secondary thickenings (Fig. 1a; Serk *et al*., 2015; Pesquet *et al*., 2019; Emonet & Hay, 2022; Blaschek *et al*., 2024).
2. The polymer three‐dimensional structure in each cell wall layer for every distinct cell type. This structure depends on the chemistries of lignin units (Boerjan *et al*., 2003), the position of these units along the lignin backbone and/or branches (Kishimoto *et al*., 2010; Mir Derikvand *et al*., 2008; Bouvier d'Yvoire *et al*., 2013; Sibout *et al*., 2005; Yamamoto *et al*., 2020), and the type of linkages between units. Together, these aspects control the spatial torsion capacity, the biomechanical and biochemical properties of each lignin polymer (Ménard *et al*., 2022).

Due to the hydric swelling/shrinking of cell wall polysaccharides (pectins, hemicelluloses and cellulose), the spatially restricted depositions of lignins act like a varnish to establish cell wall portions/areas with high susceptibility to hydration‐dependent changes (when unlignified) and with low susceptibility to hydration‐dependent changes (when lignified). The covering of cell wall polysaccharide surfaces and void spaces with lignins will still enable the formation of pores, whose dimensions and density directly depend on lignin amount and chemistry, as shown when blending different lignins in composite biomaterials (Beaucamp *et al*., 2019). Iso‐ to anisotropic cell wall deformation depending on water impregnation and/or loss in these lowly lignified areas will also depend on the orientation of cellulose microfibrils and the chemistries of their associated matrix polysaccharides, also differing within and between cell wall layers and cell types. Lignins with specific localisations, compositions and structures will thus act as:

1. Impermeable sealants for the apoplastic space to prevent free diffusion of water‐soluble compounds between cells. This is observed in endodermal cells with Casparian strips in roots (Naseer *et al*., 2012; Lee *et al*., 2013; Calvo‐Polanco *et al*., 2021), in exodermal cells with a polar lignin cap (Manzano *et al*., 2025), and between gland cells and stalk cells with ‘neck strips’ in glandular trichomes (Hao *et al*., 2024).
2. Varnishing to set the hydration‐dependent mechanical properties of xylem fibre cell walls in response to developmental and environmental conditions (Hiraide *et al*., 2021; Blaschek *et al*., 2023).
3. Mechanical adjustment to set either the reversible deformation of xylem water‐conducting tracheary elements in response to variations in water availability during plant growth and environmental changes (Ménard *et al*., 2022) or the irreversible deformation during dry fruit dehiscence for releasing/catapulting seeds (Liljegren *et al*., 2000; Perez *et al*., 2022).
4. Secured abscission zones during organ separation (Lee *et al*., 2018).
5. Gas‐permeation barriers, such as those setting microaerobic conditions to protect *Frankia* nitrogenase activity in certain actinorhizal nodules (Berg & McDowell, 1988; Schubert *et al*., 2013).
6. Mechano‐chemical protection barriers against pathogens and herbivore damages (Grabber *et al*., 1998; Dashtban *et al*., 2010; Skyba *et al*., 2013; Lee *et al*., 2019; Cao *et al*., 2022; Huang *et al*., 2023).
7. Oxidation protection barriers against abiotic factors, including ionising radiation, ultraviolet (UV) light and reactive oxygen species (ROS) (Sadeghifar & Ragauskas, 2020; Lin *et al*., 2021; Tran *et al*., 2021).

## III. The controlled combinatorial assembly of lignin polymers

**Fig. 2:**
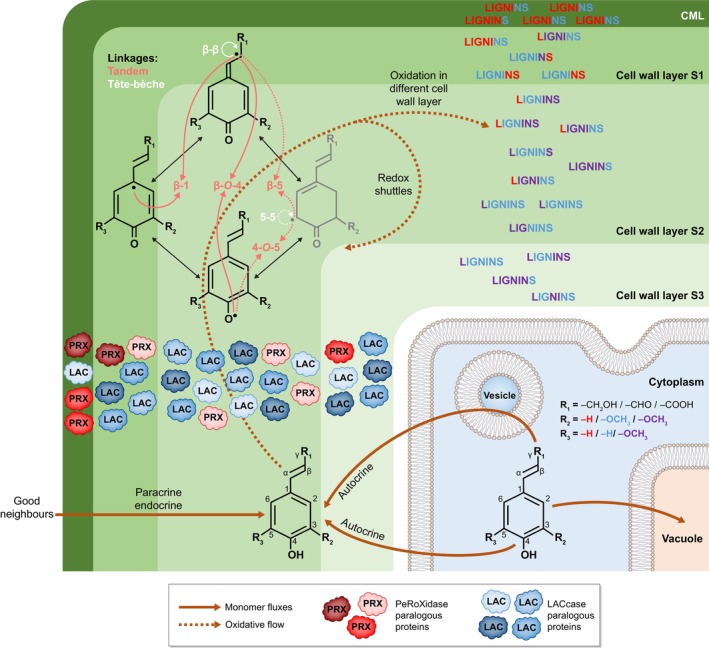
Lignins are spatially regulated by differences in the oxidative capacities of each cell wall layer in each cell type to adjust to growth and/or environmental changes. Schematic representation of the cellular and molecular mechanisms controlling the accumulation of specific lignin topochemistries in plant cell walls. These mechanisms involve the biosynthesis of lignin precursors in the cytoplasm from aromatic amino acids (mainly phenylalanine but also tyrosine) into C_6_C_3_ phenylpropanoids with different ring substitutions and different terminal aliphatic functions (differently colour‐coded for each unit with H ringed units in red, G in blue and S in purple), their transport across the plasma membrane to the cell wall either cell‐autonomously (autocrine) or cooperatively (para‐ and/or endocrine depending on transit through the xylem sap) represented by large brown plain arrows, and their oxidation into resonating radicals that can cross‐couple into various tandem or *tête‐bèche* linkages indicated by large brown dotted arrows. The chemical diversity of lignin monomers is shown by the R_1_ group (either alcohol ‐CH_2_OH, aldehyde ‐CHO or carboxylic acid –COOH) and the R_2_ and R_3_ groups (either –H or –OCH_3_). Note that tandem (in red) or *tête‐bèche* (in white) linkages possible for C_6_C_3_ phenylpropanoids with at least one unsubstituted *meta* position are indicated by dotted lines. The different lignin oxidative capacities of each cell wall layer – primary cell wall layers forming the compound middle lamella (CML) and secondary cell wall layers S1 to S3 – present different stoichiometries of LACCASEs (LACs) and/or PEROXIDASEs (PRXs) paralogous isozyme combinations for controlling local lignin topochemistry. The resulting lignin polymers are indicated as ‘LIGNINS’ in the different cell wall layers, with composition according to the unit colour code (only considering aromatic ring substitution) and concentrations depending on the number of words per cell wall layer thickness.

Lignin monomers are C_6_C_X_ phenyl‐substituted compounds varying in their C_6_ *meta* and *para* substitution levels, in their aliphatic terminal function, in their aliphatic length C_X_ (with X ranging from 1 to 9 carbons) and in the proportion of unsaturated bonds in their aliphatic chains (Box 1; Table 1; Fig. 2). The carbon positions are generally labelled from 1 to 6 in the C_6_ ring and from α to ω or from 7 and beyond in the aliphatic C_X_ chain (Boerjan *et al*., 2003). The most abundant monomers are C_6_C_3_ phenylpropanoids with C_6_ *para* hydroxyl and aliphatic unsaturated C_3_ with an alcohol terminal function, also called monolignols. The biosynthesis of lignin C_6_C_3_ monomers is made at the interface between the outer surface of the endoplasmic reticulum and the cytoplasm (Gou *et al*., 2018). Aromatic amino acids formed by the plastidial shikimate pathway – phenylalanine in all plant species as well as tyrosine in some monocot species – are modified into distinct C_6_C_3_ phenylpropanoids (Fig. 2; Boerjan *et al*., 2003; Barros *et al*., 2016). Other phenolic compounds derived from intermediates of the plastidial shikimate pathway and/or the cytoplasmic phenylpropanoid pathway (Table 1) are also naturally incorporated into lignins of specific cell types in response to developmental and/or environmental constraints to further diversify lignin properties. These compounds include C_6_C_1_ phenylmethanoids like *p*‐hydroxybenzoic acid in *Posidonia* and poplar (Vanholme *et al*., 2012; Kaal *et al*., 2018; Hu *et al*., 2022; Mottiar *et al*., 2023a), C_6_C_9_ flavonoids such as tricin in grasses, vanilla and *Medicago* (Lan *et al*., 2015; Liu *et al*., 2022; Lin *et al*., 2021), amentoflavone in *Selaginella* (Rencoret *et al*., 2021) and naringenin in papyrus (Rencoret *et al*., 2022), or dimers linked by ester or amide bonds like feruloyl‐tyramine in tomato in response to stem‐boring herbivores (Kashyap *et al*., 2022) or in the periderm of potato tubers (del Río *et al*., 2025).

**Table 1:** List of the 49 currently known lignin units detected to accumulate in lignins.

C_6_ ring	C_6_ *para* 4	C_6_ *meta* 3	C_6_ *meta* 5	C_X_ aliphatic	Aliphatic function	Lignomic/HSQC terminology	Monomer name	Occurrence	References
P	‐H	‐H	‐H	C_1_	Ester (of sinapate)		Benzoate*	Dicots (Populus spp), Monocots (Palms)	Karlen *et al*. (2017), Rencoret *et al*. (2018)
H	‐OH	‐H	‐H	C_1_	Aldehyde	V^6^	*p*‐Hydroxybenzaldehyde	Conifers, Dicots (Populus spp)	Ralph *et al*. (1997), Kim *et al*. (2003)
	‐OH	‐H	‐H	C_1_	Ester	PB^2^	*p*‐Hydroxybenzoyl ester	Dicots (Populus spp)	Mansfield *et al*. (2012)
	‐OH	‐H	‐H	C_1_	Acid	VA^6^	*p*‐Hydroxybenzoate	Dicots (beech, birch)	Smit *et al*. (2022)
	‐OH	‐H	‐H	C_3_	Ester of acetate	pCA^2^/pCE^3^	*p*‐Coumaryl acetate	Monocots, Dicots	Ralph (1996), Lu & Ralph (2002), del Río *et al*. (2007), Karlen *et al*. (2017)
	‐OH	‐H	‐H	C_3_	Ester of *p*‐coumarate	pCA^2^/pCE^3^	*p*‐Coumaroyl coumarate	Monocots	Smith (1955a), Higuchi *et al*. (1967), Karlen *et al*. (2017), Karlen *et al*. (2018)
	‐OH	‐H	‐H	C_3_	Acid	pCA^8^	*p*‐Coumaric acid	Monocots, Dicots	Smith *et al*. (2022)
	‐OH	‐H	‐H	C_3_	Aldehyde		*p*‐Coumaraldehyde	Cucurbitaceae	Varbanova *et al*. (2011)
	‐OH	‐H	‐H	C_3_	Alcohol	H^1^	*p*‐Coumaryl alcohol	Tracheophytes	Freudenberg & Neish (1968)
	‐OH	‐H	‐H	C_7_	di‐*meta‐*hydroxyphenylethene		Resveratrol	Commelinid Monocots, Norway Spruce	del Río *et al*. (2017), Rencoret *et al*. (2018), Rencoret *et al*. (2019), Neiva *et al*. (2020)
	‐OH	‐H	‐H	C_9_O	Benzopyran‐4‐one		Naringenin and naringenin chalcone	Selaginella (Lycophyte), Cycad (Gymnosperm), Cyperaceae (Monocot)	Rencoret *et al*. (2021), Rencoret *et al*. (2022)
C	‐OH	‐OH	‐H	C_3_	Alcohol	C^7^ – C_6_ terminal: C′^7^	Caffeyl alcohol	Monocots (Orchidaceae), Dicots (Cleomaceae, Euphorbiaceae, Cactaceae)	Chen *et al*. (2012), Chen *et al*. (2013), Tobimatsu *et al*. (2013)
	‐OH	‐OH	‐H	C_8_	di‐*meta‐*hydroxyphenylethene		Piceatannol	Commelinid Monocots	del Río *et al*. (2017), Rencoret *et al*. (2018), Rencoret *et al*. (2019), Neiva *et al*. (2020)
G	‐OH	‐OCH_3_	‐H	C_1_	Acid		Vanillate	Dicots (Arabidopsis spp)	Eudes *et al*. (2012)
	‐OH	‐OCH_3_	‐H	C_1_	Aldehyde	C_3_ terminal: X3^10^	Vanillin	Conifers, Dicots (Populus spp)	Ralph *et al*. (1997), Kim *et al*. (2003)
	‐OH	‐OCH_3_	‐H	C_3_	Ester of acetate	C_3_ terminal: I′^3^	Coniferyl acetate	Monocots, Dicots	Ralph (1996), Lu & Ralph (2002), del Río *et al*. (2007), Karlen *et al*. (2017)
	‐OH	‐OCH_3_	‐H	C_3_	Ester of benzoate	C_3_ terminal: I′^3^	Coniferyl benzoate	Monocots (Arecaceae)	Karlen *et al*. (2017)
	‐OH	‐OCH_3_	‐H	C_3_	Ester of *p*‐hydroxybenzoate	C_3_ terminal: I′^3^	Coniferyl *p*‐hydroxybenzoate	Monocots (Arecaceae, Posidoniaceae), Dicots (Araliaceae, Salicaceae, Saxifragaceae)	Smith (1955a,b), Hibino *et al*. (1994), Kaal *et al*. (2018), Faleva *et al*. (2020)
	‐OH	‐OCH_3_	‐H	C_3_	Ester of vanillate	C_3_ terminal: I′^3^	Coniferyl vanillate	Monocots (Arecaceae)	Karlen *et al*. (2017)
	‐OH	‐OCH_3_	‐H	C_3_	Ester of *p*‐coumarate	C_3_ terminal: I′^3^	Coniferyl *p*‐coumarate	Monocots, Dicots (Rosales, kenaf)	Smith (1955a), Higuchi *et al*. (1967), Mottiar *et al*. (2023b), Hellinger *et al*. (2023), Hellinger *et al*. (2025)
	‐OH	‐OCH_3_	‐H	C_3_	Ester of ferulate	FA^2^ – C_3_ terminal: I′^3^	Coniferyl ferulate	Monocots, Dicots	Karlen *et al*. (2016), Hellinger *et al*. (2025)
	‐OH	‐OCH_3_	‐H	C_3_	Acid	FA^8^	Ferulate	Dicots	Ralph *et al*. (2008)
	‐OH	‐OCH_3_	‐H	C_3_	Aldehyde	G'^1^ – C_3_ terminal: X2^4^/J^3^	Coniferaldehyde	Spermatophytes	Adler & Ellmer (1948), Pillonel *et al*. (1991), Halpin *et al*. (1994), Baucher *et al*. (1996), Ralph *et al*. (1997), Sibout *et al*. (2005)
	‐OH	‐OCH_3_	‐H	C_3_	Alcohol	G^1^ – C_3_ terminal: X1^2^/I^3^	Coniferyl alcohol	Tracheophytes	Freudenberg & Neish (1968)
	‐OH	‐OCH_3_	‐H	Propanal C_3_	Alcohol		Dihydroconiferyl alcohol	Conifers	Ralph *et al*. (1997)
	‐OH	‐OCH_3_	‐H	Propane 1,3‐diol C_3_	Alcohol		Arylpropane‐1,3‐diol	Conifers	Ralph *et al*. (1999a,b)
	‐OH	‐OCH_3_	‐H	Glycerol C_3_	Glycerol	C_3_ terminal:X^8^	Guaiacylglycerol	Dicots (Medicago spp)	Ralph *et al*. (2006)
	‐OH	‐OCH_3_	‐H	C_3_	Amide (of phenylethane)	Ft^9^	Feruloyl tyramine	Dicots (Nicotiana spp)	Joo *et al*. (2021)
	‐OH	‐OCH_3_	‐H	C_3_	Amide (of feruloylputrescine)		Diferuloyl putrescine	Monocots (Zea spp)	del Río *et al*. (2018)
	‐OH	‐OCH_3_	‐H	C_3_	Amide (of octopamine)	Fo^9^	Feruloyl octopamine	Dicots (Solenacea)	del Río *et al*. (2025)
	‐OH	‐OCH_3_	‐H	C_7_	di‐*meta‐*hydroxyphenylethene		Isorhapontigenin	Conifers	Rencoret *et al*. (2019), Neiva *et al*. (2020)
5H	‐OH	‐OCH_3_	‐OH	C_3_	Aldehyde	5H′^1^, MC′^3^	5‐Hydroxyconiferaldehyde	Monocots, Dicots	Morrell *et al*. (2010)
	‐OH	‐OCH_3_	‐OH	C_3_	Alcohol	5H^1^, MC^3^	5‐Hydroxyconiferyl alcohol	Monocots, Dicots	Atanassova *et al*. (1995), Suzuki *et al*. (1997), Van Doorsselaere *et al*. (1995), Do *et al*. (2007), Chen *et al*. (2013)
S	‐OH	‐OCH_3_	‐OCH_3_	C_1_	Acid	S"^5^	Syringate	Dicots (Arabidopsis spp)	Eudes *et al*. (2012)
	‐OH	‐OCH_3_	‐OCH_3_	C_1_	Aldehyde	SA^6^ – C_3_ terminal: X3^10^	Syringaldehyde	Conifers, Dicots (Arabidopsis)	Kim *et al*. (2003), Eudes *et al*. (2012)
	‐OH	‐OCH_3_	‐OCH_3_	C_3_	Ester of acetate	C_3_ terminal: I′^3^	Sinapoyl acetate	Monocots, Dicots	Ralph (1996), Lu & Ralph (2002), del Río *et al*. (2007), Karlen *et al*. (2017)
	‐OH	‐OCH_3_	‐OCH_3_	C_3_	Ester of benzoate	C_3_ terminal: I′^3^	Sinapoyl benzoate	Monocots (Arecaceae)	Karlen *et al*. (2017)
	‐OH	‐OCH_3_	‐OCH_3_	C_3_	Ester of *p*‐hydroxybenzoate	C_3_ terminal: I′^3^	Sinapoyl *p*‐hydroxybenzoate	Monocots (Arecaceae, Posidoniaceae), Dicots (Araliaceae, Salicaceae, Saxifragaceae)	Smith (1955a,b), Hibino *et al*. (1994), Kaal *et al*. (2018), Faleva *et al*. (2020)
	‐OH	‐OCH_3_	‐OCH_3_	C_3_	Ester of vanillate	C_3_ terminal: I′^3^	Sinapoyl vanillate	Monocots (Arecaceae)	Karlen *et al*. (2017)
	‐OH	‐OCH_3_	‐OCH_3_	C_3_	Ester of *p*‐coumarate	C_3_ terminal: I′^3^	Sinapoyl *p*‐coumarate	Monocots, Dicots (Rosales, kenaf)	Smith (1955a,b), Higuchi *et al*. (1967), Mottiar *et al*. (2023b), Hellinger *et al*. (2023), Hellinger *et al*. (2025)
	‐OH	‐OCH_3_	‐OCH_3_	C_3_	Ester of ferulate	C_3_ terminal: I′^3^	Sinapoyl ferulate	Monocots, Dicots	Karlen *et al*. (2016)
	‐OH	‐OCH_3_	‐OCH_3_	C_3_	Acid		Sinapate	Dicots	Ralph *et al*. (2008)
	‐OH	‐OCH_3_	‐OCH_3_	C_3_	Aldehyde	S'^1^ – C_3_ terminal: X2^4^/J^3^	Sinapaldehyde	Spermatophytes	Adler & Ellmer (1948), Pillonel *et al*. (1991), Halpin *et al*. (1994), Baucher *et al*. (1996), Ralph *et al*. (1997), Sibout *et al*. (2005)
	‐OH	‐OCH_3_	‐OCH_3_	C_3_	Alcohol	S^1^ ‐ C_3_ terminal: X1^2^/I^3^	Sinapyl alcohol	Selaginellaceae, Gnetophyta, Angiosperms	Freudenberg & Neish (1968)
	‐OH	‐OCH_3_	‐OCH_3_	Glycerol C_3_	Glycerol	C_3_ terminal:X^8^	Syringyl glycerol	Dicots (Medicago spp)	Ralph *et al*. (2006)
	‐OH	‐OCH_3_	‐OCH_3_	C_3_	Acetate		Sinapyl acetate	Monocots, Dicots	Karlen *et al*. (2017), Rencoret *et al*. (2022)
	‐OH	‐OCH_3_	‐OCH_3_	C_9_O	Benzopyran‐4‐one	T^3^	Tricin and dihydrotricin	Monocots, Dicots (Medicago spp)	del Río *et al*. (2012), Lan *et al*. (2016), Rencoret *et al*. (2022)

Units are classified according to (1) their C_6_ aromatic ring substitution (grouped in grey or white to show similar ring chemistry), (2) the carbon length of their C_X_ aliphatic chain and (3) the terminal aliphatic function. Differences in C_6_ aromatic rings include nonsubstituted phenyl (P), *para* hydroxylated *p*‐hydroxyphenyl (H), *para/meta* hydroxylated caffeyl (C), *para* hydroxylated and *meta* methoxylated guaiacyl (G), *para/meta* hydroxylated and *meta* methoxylated 5‐hydroxyguaiacyl (5H) and *para* hydroxylated and *meta/meta* methoxylated syringyl (S). The originating monomers, as well as the occurrence/distribution in plants and references, are listed for each unit. Correspondence to previous lignomics and HSQC terminology is also provided for unit presence, as well as position (indicated by terminal either for free aliphatic chain with C_3_ terminal or for free aromatic ring with C_6_ terminal) with ^1^taken from Morreel *et al*. (2010); ^2^from Mansfield *et al*. (2012); ^3^from Wen *et al*. (2013); ^4^from Kim *et al*. (2017); ^5^from Zhao *et al*. (2017); ^6^from Smit *et al*. (2022); ^7^from Tobimatsu *et al*. (2013); ^8^from Krivdin (2025); ^9^from del Río *et al*. (2025); and ^10^from Perez *et al*. (2022). * Note that benzoate lacks a *para* hydroxyl group and is incorporated as an ester of phenylpropanoid.

The key chemical feature for a compound to be incorporated as a lignin unit is the presence of a C_6_ *para* hydroxyl that can be oxidised to enable the end‐wise extension of lignin polymers (Fig. 2; Boerjan *et al*., 2003; Vanholme *et al*., 2012). Lignins apparently restrictively incorporate monomers with diametrically positioned (or *para*) hydroxyl and aliphatic groups. Other phenolic compounds such as salicylic acid, with an *ortho* positioned hydroxyl group to the aliphatic chain, although transported in the sap of lignified xylem (Ratzinger *et al*., 2009) or in exosomes of lignifying cell suspensions (Kankaanpää *et al*., 2024) have not been detected in lignins, although salicylate can be oxidised and dimerised by phenoloxidases (such as LACCASEs (LACs); Ciecholewski *et al*., 2005). The chemical diversification among monomers will depend on the C_6_ *meta* groups and on the aliphatic C_X_. Differences in C_6_ *meta* groups distinguish rings with no substitution, called *p‐*hydroxyphenyl (H), from those with one hydroxyl group in the *meta* position, called caffeyl (C), with one *meta* methoxy group, called guaiacyl (G), with one *meta* hydroxyl and one methoxy groups, called 5‐hydroxyguaiacyl (5H), and with two methoxy groups, called syringyl (S) (Table 1).

C_6_C_x_ compounds can also serve as radical redox shuttle mediators that, once oxidised, transfer the radical charge to other redox compatible compounds, allowing/optimising their incorporation into the growing lignin polymers (Nishimoto *et al*., 2024). Although only shown *in vitro*, phenolic compounds such as vanillin, acetovanillone, syringaldehyde, methylsyringate, acetosyringone, *p*‐coumarate and ferulate can effectively act as both mediators and monomers (Cañas & Camarero, 2010; Chen *et al*., 2021; Smith *et al*., 2022; Martin *et al*., 2024). The natural plasticity in lignification allows the incorporation of other compounds with C_6_ *para* hydroxyl not normally present in lignins. For instance, the targeted transgenic overproduction in lignified tissues of curcumin/C_6_C_7_C_6_ or scopoletin/C_6_C_3_ enables their incorporation into lignins, either by direct oxidation by phenoloxidases or by indirect activation by redox shuttles (Oyarce *et al*., 2019; De Meester *et al*., 2022; Hoengenaert *et al*., 2022). This chemical diversity in C_6_ and C_x_ provides each lignin monomer with only partly redundant functions on lignin polymers (Tables 1, 2). Beside changes in interlinkage type capacity (Table 2), these chemical differences enable specific units to have unique properties, including:

1. Terminating properties promoting the unidirectional extension of lignin polymers such as for tricin S C_6_C_9_ units (Lan *et al*., 2015, 2016) that are restrictively present at the polymer termini, as their aliphatic part cannot form linkages (note that tricin is incorporated as the initiating monomer in oligo/polymers; Table 2).
2. Linearising properties with units favouring the formation of only one preferential type of linkage without branching, such as benzodioxane (α‐*O‐*5/β‐*O‐*4) linkages in‐between units with C_6_ catechol ring substitutions such as C and 5H (Chen *et al*., 2012; Tobimatsu *et al*., 2013; S. Wang *et al*., 2022).
3. Stretching properties by limiting the lignin polymers capacity to fold onto itself, such as with internal C_6_C_3_ units with terminal aldehyde functions linked by β‐*O‐*4 (Ménard *et al*., 2022).

**Table 2:** List of the accessible atoms for linkage possibilities and unit position of 49 currently known lignin units if supplied as monomers.

C_6_ ring	C_6_ *para* 4	C_6_ *meta* 3	C_6_ *meta* 5	C_X_ aliphatic	Monomer name	Available positions for linkages								Unit position in polymer		
						C6‐O	C6‐5	C6‐3	C3‐α	C3‐β	C3‐γ	C3‐ε	C3‐θ	C_3_ terminal	C_6_ terminal	Internal
P	‐H	‐H	‐H	C_1_	Benzoate*	No	No	No	No	n/a	n/a	n/a	n/a	No	Yes	No
H	‐OH	‐H	‐H	C_1_	*p*‐Hydroxybenzaldehyde	Yes	Yes	Yes	No	n/a	n/a	n/a	n/a	Yes	Yes	Yes
	‐OH	‐H	‐H	C_1_	*p*‐Hydroxybenzoyl ester	Yes	Yes	Yes	No	n/a	n/a	n/a	n/a	Yes	Yes	Yes
	‐OH	‐H	‐H	C_1_	*p*‐Hydroxybenzoate	Yes	Yes	Yes	No	n/a	n/a	n/a	n/a	Yes	Yes	Yes
	‐OH	‐H	‐H	C_3_	*p*‐Coumaryl acetate	Yes	Yes	Yes	Yes	Yes	No	n/a	n/a	Yes	Yes	Yes
	‐OH	‐H	‐H	C_3_	*p*‐Coumaroyl coumarate	Yes^2^	Yes^2^	Yes^2^	Yes^2^	Yes^2^	No	n/a	n/a	Yes	Yes	Yes
	‐OH	‐H	‐H	C_3_	*p*‐Coumaric acid	Yes	Yes	Yes	Yes	Yes	No	n/a	n/a	Yes	Yes	Yes
	‐OH	‐H	‐H	C_3_	*p*‐Coumaraldehyde	Yes	Yes	Yes	Yes	Yes	No	n/a	n/a	Yes	Yes	Yes
	‐OH	‐H	‐H	C_3_	*p*‐Coumaryl alcohol	Yes	Yes	Yes	Yes	Yes	No	n/a	n/a	Yes	Yes	Yes
	‐OH	‐H	‐H	C_7_	Resveratrol^b^	Yes^1^	Yes^1^	Yes^1^	Yes^1^	Yes^1^	No	Yes^1^	Yes^1^	Yes	Yes	Yes
	‐OH	‐H	‐H	C_9_O	Naringenin chalcone	Yes	Yes	Yes	No	Yes	No	No	No	Yes	Yes	Yes
	‐OH	‐H	‐H	C_9_O	Naringenin	Yes	Yes	Yes	No	No	No	No	No	Yes	Yes	Yes
C	‐OH	‐OH	‐H	C_3_	Caffeyl alcohol^a^	Yes	Yes	No	Yes	Yes	No	n/a	n/a	Yes	Yes	Yes
	‐OH	‐OH	‐H	C_8_	Piceatannol^a,b^	Yes^1^	Yes^1^	No	Yes^1^	Yes^1^	No	Yes^1^	Yes^1^	Yes	Yes	Yes
G	‐OH	‐OCH_3_	‐H	C_1_	Vanillate	Yes	Yes	No	No	n/a	n/a	n/a	n/a	Yes	Yes	Yes
	‐OH	‐OCH_3_	‐H	C_1_	Vanillin	Yes	Yes	No	No	n/a	n/a	n/a	n/a	Yes	Yes	Yes
	‐OH	‐OCH_3_	‐H	C_3_	Coniferyl acetate	Yes	Yes	No	No	n/a	n/a	n/a	n/a	Yes	Yes	Yes
	‐OH	‐OCH_3_	‐H	C_3_	Coniferyl benzoate	Yes^1^	Yes^1^	No	Yes^1^	Yes^1^	No	n/a	n/a	Yes	Yes	Yes
	‐OH	‐OCH_3_	‐H	C_3_	Coniferyl *p*‐hydroxybenzoate	Yes^2^	Yes^2^	Yes^1^	Yes^1^	Yes^1^	No	n/a	n/a	Yes	Yes	Yes
	‐OH	‐OCH_3_	‐H	C_3_	Coniferyl vanillate	Yes^2^	Yes^2^	No	Yes^1^	Yes^1^	No	n/a	n/a	Yes	Yes	Yes
	‐OH	‐OCH_3_	‐H	C_3_	Coniferyl *p*‐coumarate	Yes^2^	Yes^2^	Yes^1^	Yes^2^	Yes^2^	No	n/a	n/a	Yes	Yes	Yes
	‐OH	‐OCH_3_	‐H	C_3_	Coniferyl ferulate	Yes^2^	Yes^2^	No	Yes^2^	Yes^2^	No	n/a	n/a	Yes	Yes	Yes
	‐OH	‐OCH_3_	‐H	C_3_	Ferulate	Yes	Yes	No	Yes	Yes	No	n/a	n/a	Yes	Yes	Yes
	‐OH	‐OCH_3_	‐H	C_3_	Coniferaldehyde	Yes	Yes	No	Yes	Yes	No	n/a	n/a	Yes	Yes	Yes
	‐OH	‐OCH_3_	‐H	C_3_	Coniferyl alcohol	Yes	Yes	No	Yes	Yes	No	n/a	n/a	Yes	Yes	Yes
	‐OH	‐OCH_3_	‐H	Propanal C_3_	Dihydroconiferyl alcohol	Yes	Yes	No	Yes	No	No	n/a	n/a	Yes	Yes	Yes
	‐OH	‐OCH_3_	‐H	Propane 1,3‐diol C_3_	Arylpropane‐1,3‐diol	Yes	Yes	No	Yes	No	No	n/a	n/a	Yes	Yes	Yes
	‐OH	‐OCH_3_	‐H	Glycerol C_3_	Guaiacylglycerol	Yes	Yes	No	Yes	No	No	n/a	n/a	Yes	Yes	Yes
	‐OH	‐OCH_3_	‐H	C_3_	Feruloyl tyramine	Yes^2^	Yes^2^	Yes^1^	No	Yes^1^	No	n/a	n/a	Yes	Yes	Yes
	‐OH	‐OCH_3_	‐H	C_3_	Feruloyl octopamine	Yes^2^	Yes^2^	Yes^1^	No	Yes^1^	No	n/a	n/a	Yes	Yes	Yes
	‐OH	‐OCH_3_	‐H	C_3_	Diferuloyl putrescine	Yes^2^	Yes^2^	No	No	Yes^2^	No	n/a	n/a	Yes	Yes	Yes
	‐OH	‐OCH_3_	‐H	C_7_	Isorhapontigenin^b^	Yes^1^	Yes^1^	No	Yes^1^	Yes^1^	No	Yes^1^	Yes^1^	Yes	Yes	Yes
5H	‐OH	‐OCH_3_	‐OH	C_3_	5‐Hydroxyconiferaldehyde^a^	Yes	Yes	No	Yes	Yes	No	n/a	n/a	Yes	Yes	Yes
	‐OH	‐OCH_3_	‐OH	C_3_	5‐Hydroxyconiferyl alcohol^a^	Yes	Yes	No	Yes	Yes	No	n/a	n/a	Yes	Yes	Yes
S	‐OH	‐OCH_3_	‐OCH_3_	C_1_	Syringate	Yes	No	No	No	n/a	n/a	n/a	n/a	Yes	No	No
	‐OH	‐OCH_3_	‐OCH_3_	C_1_	Syringaldehyde	Yes	No	No	No	n/a	n/a	n/a	n/a	Yes	No	No
	‐OH	‐OCH_3_	‐OCH_3_	C_3_	Sinapoyl acetate	Yes	No	No	Yes	Yes	No	n/a	n/a	Yes	Yes	Yes
	‐OH	‐OCH_3_	‐OCH_3_	C_3_	Sinapoyl benzoate	Yes^1^	No	No	Yes^1^	Yes^1^	No	n/a	n/a	Yes	Yes	Yes
	‐OH	‐OCH_3_	‐OCH_3_	C_3_	Sinapoyl *p*‐hydroxybenzoate	Yes^2^	Yes^1^	Yes^1^	Yes^1^	Yes^1^	No	n/a	n/a	Yes	Yes	Yes
	‐OH	‐OCH_3_	‐OCH_3_	C_3_	Sinapoyl vanillate	Yes^2^	Yes^1^	No	Yes^1^	Yes^1^	No	n/a	n/a	Yes	Yes	Yes
	‐OH	‐OCH_3_	‐OCH_3_	C_3_	Sinapoyl *p*‐coumarate	Yes^2^	Yes^1^	Yes^1^	Yes^2^	Yes^2^	No	n/a	n/a	Yes	Yes	Yes
	‐OH	‐OCH_3_	‐OCH_3_	C_3_	Sinapoyl ferulate	Yes^2^	Yes^1^	No	Yes^2^	Yes^2^	No	n/a	n/a	Yes	Yes	Yes
	‐OH	‐OCH_3_	‐OCH_3_	C_3_	Sinapate	Yes	No	No	Yes	Yes	No	n/a	n/a	Yes	Yes	Yes
	‐OH	‐OCH_3_	‐OCH_3_	C_3_	Sinapaldehyde	Yes	No	No	Yes	Yes	No	n/a	n/a	Yes	Yes	Yes
	‐OH	‐OCH_3_	‐OCH_3_	C_3_	Sinapyl alcohol	Yes	No	No	Yes	Yes	No	n/a	n/a	Yes	Yes	Yes
	‐OH	‐OCH_3_	‐OCH_3_	Glycerol C_3_	Syringyl glycerol	Yes	No	No	Yes	No	No	n/a	n/a	Yes	Yes	Yes
	‐OH	‐OCH_3_	‐OCH_3_	C_3_	Sinapyl acetate	Yes	No	No	Yes	No	No	n/a	n/a	Yes	Yes	Yes
	‐OH	‐OCH_3_	‐OCH_3_	C_9_O	Dihydrotricin	Yes	No	No	No	No	No	No	No	Yes	No	No
	‐OH	‐OCH_3_	‐OCH_3_	C_9_O	Tricin^c^	Yes	No	No	No	Yes	No	No	No	Yes	No	No

Differences in C_6_ aromatic ring include nonsubstituted phenyl (P), *para* hydroxylated *p*‐hydroxyphenyl (H), *para/meta* hydroxylated caffeyl (C), *para* hydroxylated and *meta* methoxylated guaiacyl (G), *para/meta* hydroxylated and *meta* methoxylated 5‐hydroxyguaiacyl (5H) and *para* hydroxylated and *meta/meta* methoxylated syringyl (S). The table has been colour‐coded to indicate possibility in blue with ‘yes’, impossibility in red with ‘no’ or otherwise n/a for nonapplicable in grey/white. Units can be positioned internally (labelled internal) or terminally either with free aliphatic chain (C_3_ terminal) or free aromatic ring (C_6_ terminal). Note that although each monomer has multiple radical delocalisation possibilities, some monomers restrictively form one type of linkage, such as α‐*O‐*5/β‐*O*‐4 benzodioxane for units with C_6_ catechol (1,2 dihydroxybenzene, labelled^a^) or α‐*O‐*ζ/β‐*ε* for units with C_6_ resorcinol (1,3 dihydroxybenzene, labelled^b^) or β‐*O*‐4 aryl ether for units with tricin (placing them always in C_3_ terminal position, labelled^c^). As some monomers display a dimeric structure linked by ester or amide bonds, each radical resonance possibility is indicated with ^1^if affecting only one part of the monomer or with ^2^if affecting both parts. *Note that benzoate lacks a *para* hydroxyl group and is incorporated as an ester of phenypropanoid.

Although each unit will have a different impact on the lignin polymer depending on its chemistry and its position, it is only recently that the different functions of chemically distinct units are becoming apparent for plant development and physiology.

Due to the dual high reactivity of *para* hydroxylated C_6_C_X_ compounds as redox shuttle mediators and lignin monomers, the regulation of their extracellular availability by plant cells will determine the extent of their contribution to lignin topochemistry. Once formed in the cytoplasm, these various monomeric precursors can be: either further conjugated into glycosyl ethers and/or esters; assembled into oligomers and stored in the vacuole (Dima *et al*., 2015) or in vesicles (Jeon *et al*., 2023; Kankaanpää *et al*., 2024); accumulate as molecular condensates (Xie *et al*., 2023); and/or be directly released into the cell wall (Fig. 2). It remains unclear what differences in the chemistries are used to distinguish *bona fide* lignin precursors from their biosynthetic intermediates and other related nonlignin phenolic end‐products (lignans, neolignans and/or oligolignols). The direct external supply of coniferyl alcohol (forming G C_6_C_3_ alcohol units) to isolated dead tracheary elements in suspension cultures, initially inhibited from producing any phenolic compounds, can fully restore lignin accumulation specifically in secondary cell walls in both *Zinnia violacea* and *Arabidopsis thaliana* (Pesquet *et al*., 2013; Van de Wouwer *et al*., 2016). However, these cell culture systems only produce 50% of tracheary elements, leaving the remaining 50% as parenchymatic cells (Pesquet *et al*., 2013; Ménard *et al*., 2024) capable of further modifying the supply of phenolic compounds, such as providing various C_6_C_3_ dimers (Tokunaga *et al*., 2005). Similarly, the external supply of coniferyl alcohol to young *Arabidopsis* seedlings inhibited to produce phenolic compounds can fully restore the lignification of the Casparian strip to reestablish a functional apoplastic barrier (Naseer *et al*., 2012). However, other surrounding cell types (also called ‘good neighbours’ enabling ‘noncell autonomous’ processes) might also supply phenolic compounds to ensure their incorporation into endodermal lignins. Radiolabelled monolignol glucosides, as well as other phenolic compounds such as benzoyl glucosides, ferulic and sinapic acids, supplied directly to plant cuttings of pine, magnolia, lilac, poplar, beech or sugarcane exhibited radioactive signals accumulating into specific lignins depending on cell wall layer, cell type and developmental conditions (Terashima *et al*., 1986, 1988; Terashima & Fukushima, 1988; Fukushima & Terashima, 1991; He & Terashima, 1991). Monolignol glucosides have been hypothesised as lignin precursors due to glycosylation that increases their solubility in water and also protects their *para* hydroxyl from any premature oxidation until freed as aglycones by β‐glucosidases (BGLU) in cell walls (Terashima *et al*., 2016, 2023). The formation of phenylglucoside links in lignin–carbohydrate complexes (LCCs) could derive from the incorporation of monolignol glucosides, which have been shown to be directly incorporated into synthetic lignins and detected in the native lignins of several plant species (Miyagawa *et al*., 2020). It is worth noting that artificial feeding experiments with glycosylated precursors cannot distinguish whether the fed compounds are directly incorporated into cell walls, whether their distinct glycosyl moieties are removed at the same rates (for instance, *Arabidopsis* BGLU45 more actively hydrolyses 4‐*O‐*glycosides of coniferyl and sinapyl alcohols, whereas BGLU46 prefers 4‐*O‐*glycosides of *p‐*coumaryl alcohol; Escamilla‐Treviño *et al*., 2006), or whether the provided compounds are metabolised by surrounding living cells (for instance, fed 4‐*O‐*glycosides of coniferyl alcohol or coniferin are partly oxidised into aldehyde derivatives in Ginkgo; Tsuji *et al*., 2005). Single *BGLU* loss‐of‐function mutants, however, show no observable defects in lignins (Chapelle *et al*., 2012; H. Wang *et al*., 2022). As most phenylpropanoid/lignin associated genes belong to multigenic families, stacked higher order mutants affecting multiple paralogues simultaneously are required for functional analysis to avoid the effects of redundancy. However, the three *BGLU* paralogues associated with the deglycosylation of monolignol glucosides in *Arabidopsis* (*BGLU45*, *BGLU46* and *BGLU47*; H. Wang *et al*., 2022) have not yet been stacked. The exact chemical form(s) in which lignin precursors are transported to the cell walls, and whether their *para* hydroxyl group has removable protections, remain unclear.

**Fig. 3:**
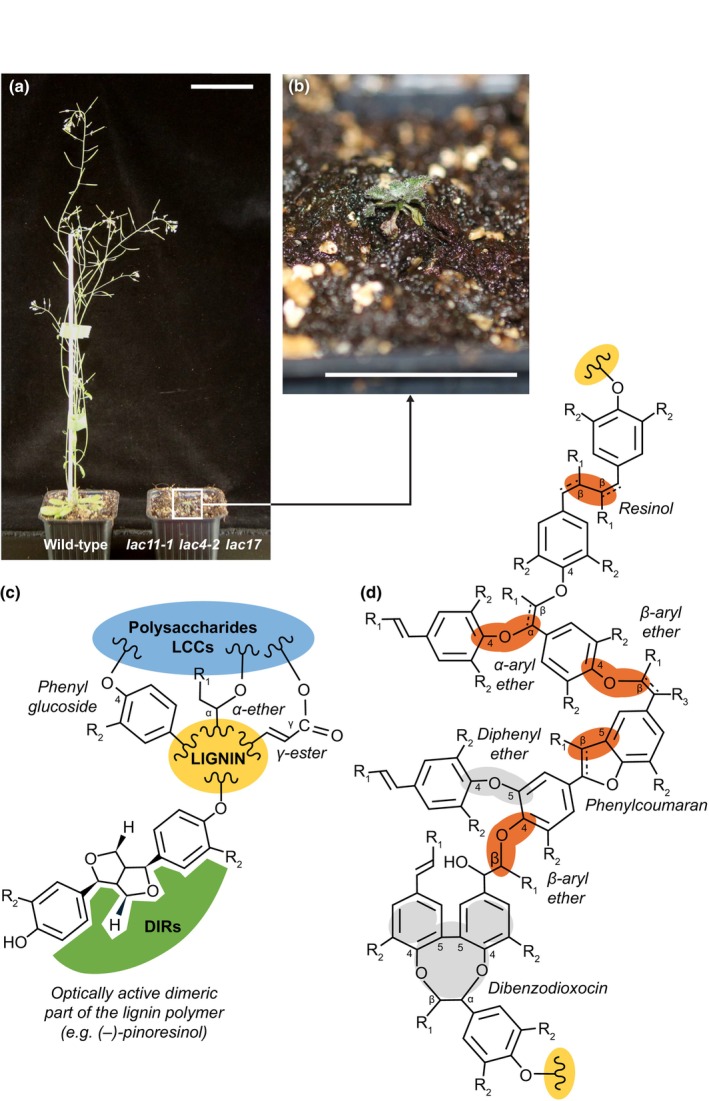
Different lignin interunit linkages are made for cell wall anchoring, initial oligomerisation and following extension. (a, b) Impact of loss of LACCASE (*LAC*)‐dependent lignins on *Arabidopsis thaliana* plant growth comparing 6‐wk‐old wild‐type (a) and *LAC* triple loss‐of‐function mutant plants (b). Bars, 4 cm (photograph taken by Dr. Emiko Murozuka). (c, d) Schematic representation of lignin molecules. (c) Lignins (indicated in yellow) are initiated and/or anchored to cell wall layers using lignin–carbohydrate complexes/LCCs (in blue), including α‐ether, γ‐ester and phenylglucoside linkages (such as through *p*‐coumarate, ferulate or tricin), and/or interacting with cell wall‐localised trimeric DIRIGENT PROTEINs/DIRs (in green) capable of binding lignin‐dimeric motives with specific optical activity. (d) Extending lignin polymers with units interlinked by β‐*O*‐4/β‐aryl ether, β‐β/resinol, β‐5/phenylcoumaran and α‐*O*‐4/α‐aryl ether (early forming linkages indicated in orange) as well as 4‐*O*‐5/diphenyl ether and 5–5/α‐*O*‐4/β‐*O*‐4/dibenzodioxocin (later forming linkages indicated in grey). The chemical diversity of lignin units is shown by the R_1_ group (either alcohol –CH_2_OH, aldehyde ‐CHO or carboxylic acid –COOH) and the R_2_ group (either –H or –OCH_3_). Note that the interunit linkage, depending on the terminal aliphatic function, can (in case of aldehydes and carboxylic acids) or not (in case of alcohols) be unsaturated as indicated by dotted lines.

Once in the apoplast, the free C_6_ *para* hydroxyl group of each monomer is oxidised to form phenoxy radicals either:

1. Nonenzymatically by UV light (Prasse *et al*., 2018) or ROS like H_2_O_2_ (Y.Q. Gao *et al*., 2023) in combination or not with ions such as Ca^2+^ or Fe^3+^/Fe^2+^ (Westermark, 1982; Minella *et al*., 2014).
2. Enzymatically by phenoloxidases, which can be either H_2_O_2_‐dependent PEROXIDASEs (PRXs) or O_2_‐dependent LACs (Figs 2, 3a; Zhao *et al.*, 2013; Rojas‐Murcia *et al.*, 2020; Blaschek & Pesquet, 2021; Blaschek *et al*., 2023).

**Fig. 4:**
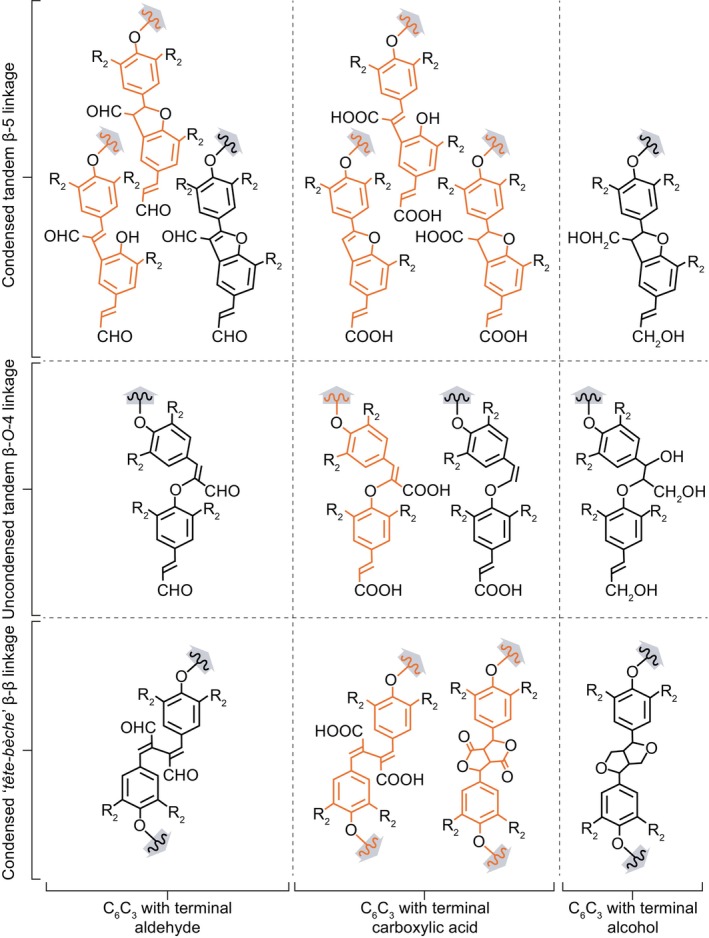
Lignin unit chemistry differently affects each interlinkage type. Schematic representation of the impact of the terminal function of C_6_C_3_ units on the structure of condensed *tête‐bèche* β‐β, condensed tandem β‐5 and uncondensed tandem β‐*O*‐4 linkages. Both aldehyde and carboxylic acid terminal functions, but not alcohols, maintain unsaturated linkages in between β‐*O*‐4 joined units (Kim *et al*., 2000; Holmgren *et al*., 2009). Condensed tandem β‐5 cyclised into phenylcoumaran cycle for units with alcohol terminal functions and in benzofuran cycles or remain uncyclised for units with aldehyde or carboxylic acid functions. While β‐β linked units with alcohol undergo resinol cyclisation, units with aldehyde do not undergo cyclisation, and carboxylic acid units may undergo cyclisation. Note that for both β‐5 and β‐*O*‐4 linkages, units with terminal carboxylic acid functions can also undergo decarboxylation. Structures presented have been identified using nuclear magnetic resonance (NMR) analyses by Ralph *et al*. (1999a); Kim *et al*. (2003); Holmgren *et al*. (2009); Matsushita *et al*. (2016) and Yoshioka *et al*. (2024). Note that lignin end‐wise extension from the free *para* hydroxyl group of the aromatic part of each unit is indicated by grey arrows, with *tête‐bèche* β‐β linkage creating inversion points promoting the bidirectional extension of lignins in contrast to the monodirectional extension of β‐5 and β‐*O*‐4 tandem linkages. Lignin structures detected only in synthetic lignins are shown in orange, whereas structures detected in both synthetic and native lignins are shown in black. The chemical diversity of lignin monomers is shown by R_2_ group (being either –H or –OCH_3_).

The formation of phenoxy radicals has been clearly shown to occur directly in the cell walls of dead wood cells (Barsberg *et al*., 2006; Blaschek *et al*., 2023). However, lignin‐like oligomers have also been identified in the lumen of vacuoles (Dima *et al*., 2015) and exosomes (Kankaanpää *et al*., 2024) together with the presence of phenoloxidases (both LACs and PRXs; Blaschek & Pesquet, 2021), indicating that the formation of phenoxy radicals also occurs in the lumen of certain organelles. Additionally, the characterisation of the cytoplasm‐localised enzyme PHENYLCOUMARAN BENZYLIC ETHER REDUCTASE in poplar, whose function is to reduce β‐5 linked phenylpropanoid dimers to form antioxidants that protect the cell against oxidative damage, further suggests intracellular formation and coupling of phenoxy radicals (Niculaes *et al*., 2014). The phenoxy radical formed by the dehydrogenation of each monomer is stabilised by resonance within its C_6_ and C_X_ structure. This delocalisation can be drawn as resonance forms, each representing the highest likelihood of encountering the radical (Fig. 2). Radicals are formed both in monomers and at the growing ends of extending oligomers by either direct oxidation by phenoloxidases or indirect radical transfer from radicalised shuttles. The probabilistic encounter of different resonance forms will lead to the coupling of the two radicals and establish the different ‐C‐C‐ and ‐C‐O‐C‐ interunit linkages found in lignins (Figs 2, 3, 4). The coupling possibilities to establish specific linkages will depend on the chemistry of each monomer, with both C_6_ substitution levels and C_X_ size affecting interunit linkage possibilities (Table 2). The relative proportion of each type of interunit linkage will also differ during the extension of lignin polymers, with a higher proportion of uncondensed β‐β linkages during initial dimerisation in contrast to their absence during further extension if not supplied as β‐β linked dimers (Hatfield & Vermerris, 2001; Matsushita *et al*., 2015). It is also clear that PRXs and LACs are not redundant and are responsible for specific lignin oxidations. For instance, oxidation of coniferyl alcohol by horseradish PRX (HRP) leads to more uncondensed lignin structure, whereas more condensed structure occurs upon its oxidation by *Trametes* sp or *Rhus vernicifera* LAC (Matsumoto *et al*., 2020; Kishimoto *et al*., 2022). The different interunit linkages will directly affect the chemical and physical properties of lignins and encompass:

1. Tandem linkages that can be repeated to concatenate units. These include: the abundant uncondensed β‐*O‐*4 linkages representing 64.8–65% of total lignin linkages in isolated tracheary elements (Wagner *et al*., 2013, 2015) or 50–65/45–50% in hard/softwood xylem tissues with mixed lignified cell types (Liu *et al*., 2022); and the less abundant β‐5, 4*‐O*‐5 and β‐1 linkages (Box 1; Figs 2, 3, 4). Note that the α‐*O*‐4 cyclisation of β‐5 linkage into phenylcoumaran or benzofuran rings depends on the terminal function of the incorporated unit (Fig. 4). Tandem linkages conserve the monodirectional orientation of extending lignin polymers with aliphatic to aromatic poles. Specific lignin units with C_6_ catechols (C and 5H) however preferentially form benzodioxane β‐*O‐*4/α‐*O*‐5 linkages (Weng *et al*., 2010; Tobimatsu *et al*., 2013). Enzymatically oxidised cyclised β‐5 structures can nevertheless resonate to open their phenylcoumaran ring at α‐*O*‐4 and offer new interunit linkage possibilities through their freed *para‐O* (Matsushita *et al*., 2019). Note that 4‐*O*‐5 linkages are rarely detected during *in vitro* lignin synthesis (Ralph *et al*., 2004; Matsushita *et al*., 2015; Tokunaga & Watanabe, 2023) or observed among the abundant oligomers in plant metabolomics analyses (Morreel *et al*., 2010; Van de Wouwer *et al*., 2016; Terrell *et al*., 2020; Kankaanpää *et al*., 2024), suggesting that these linkages might be specific to the extension of long lignin polymers (representing from 6 to 9% of all non‐β‐*O‐*4 lignin linkages in conifer wood; Li *et al*., 2016; Yue *et al*., 2016).
2. ‘*tête‐bêche*’ (tɛt.bɛ∫) inverted symmetrical linkages in‐between the same carbon position either C_6_‐to‐C_6_ (5–5) or C_3_‐to‐C_3_ (β‐β) of two consecutive units (Box 1; Figs 2, 3, 4). *Tête‐bêche* linkages inverse lignin orientation and set bidirectional foci providing new extension possibilities, either within one extending lignin chain for β‐β or between two lignin chains for 5–5 (Matsushita *et al*., 2015; Ralph *et al*., 2019). The α‐*O*‐γ cyclisation of β‐β linkage into resinol rings depends on the terminal function of the linked units (Fig. 4). Note that metabolomics analyses of oligolignols in multiple plant genera (*Arabidopsis* – Morreel *et al*., 2010; Van de Wouwer *et al*., 2016; poplar – Saleme *et al*., 2017; Terrell *et al*., 2020; *Picea –* Laitinen *et al*., 2017; Kankaanpää *et al*., 2024; sorghum – Ferreira *et al*., 2022; maize – Liu *et al*., 2021) revealed that β‐β linkages are preferentially formed during the initial dimerisation of lignin, whereas 5–5 linkages are only detected in longer lignin polymers. Many 5–5 linkages participate in the formation of the cyclic trimeric structure 5–5/β‐*O*‐4/α‐*O*‐4 dibenzodioxocin (also called ‘mickey mouse’ linkage – Fig. 3; Argyropoulos *et al*., 2002; Kukkola *et al*., 2004).
3. Linkages as ‘unextendable graft’ such as the α‐*O*‐4 linkage that only occurs when a phenoxy radical is used instead of water during β‐*O*‐4 linkage formation in between C_6_C_3_ alcohol units. The α‐*O*‐4 linkages leave their C_3_ aliphatic function free (Fig. 3). These α‐*O*‐4 linkages have not been detected among the abundant oligomers in plant metabolomics analyses (Morreel *et al*., 2010; Van de Wouwer *et al*., 2016; Terrell *et al*., 2020; Kankaanpää *et al*., 2024) but can be made *in vitro* (Saake *et al*., 1996).

Lignins contrast with other biopolymers as their accumulation in plant cell walls during growth and environmental response is irreversible, since no genes encoding lytic enzymes cleaving the different linkages between lignin units have yet been identified in plant genomes. Additional modifications of the assembled lignin polymers have also been poorly described and are apparently minor, with oxidation of C_3_ terminal alcohol units into aldehydes by PRXs (Holmgren *et al*., 2009) or addition of γ‐esterified H C_6_C_3_ acid units by LACs and redox mediators (Hilgers *et al*., 2020). The formation and functions of these other modifications on lignins still remain unclear.

## IV. Topochemical control of lignin formation

Lignin concentration, structure and chemistry differ greatly between the different cell wall layers of each cell type during their maturation and in response to environmental changes (Barros *et al*., 2015; Pesquet *et al*., 2019). This spatio‐temporal control of lignin concentration, structure and chemistry depends on differences between the redox potentials of each distinct C_6_C_X_, the concentration of phenolic monomers available in the cell wall at a given time and the oxidative capacity of the cell wall layer/site. The spatial control of lignin concentration in specific cell wall layers has been selected during evolution and speciation to respond to both developmental and environmental constraints (Blaschek *et al*., 2024). Some lignin accumulation sites are conserved between distantly related plant species, such as the levels of lignin concentration between primary and secondary cell walls of tracheary elements from lycophytes to angiosperms (Boyce *et al*., 2004). Conversely, some angiosperms also display drastic differences in lignin concentration when comparing cell wall layers, including nearly lignin‐free primary cell walls of tracheary elements in eastern leatherwood *Dirca palustris* (Mottiar *et al*., 2020) or nearly lignin‐free secondary cell walls of tracheary elements in the seagrass *Zostera marina* (Pfeifer *et al*., 2022). Even within one plant species, lignin concentration varies in tracheary elements during development (Ménard *et al*., 2022), between morphotypes in vascular tissues (Blaschek *et al*., 2023), and also between tissues of the same organ, such as in gymnosperm needles between vascular and transfusion tracheids (C. Gao *et al*., 2023; Mai *et al*., 2024). Under environmental constraints, tracheids in conifers change the distribution of their secondary cell wall lignins from an even distribution in S1 to S3 layers under normal conditions to an uneven over‐accumulation in S2 under gravitropic constraints (Fukushima & Terashima, 1991; Hiraide *et al*., 2021). These changes in lignin concentrations coincided with changes in phenoloxidase paralogue numbers between plant species, more specifically with larger reduction in LACs than in PRXs for aquatic *Zostera* compared to land plants (Simões *et al*., 2020). The presence of specific LAC paralogous isozymes also coincides with the accumulation of lignins in specific cell wall layers like AtLAC12 for the primary cell walls between interfascicular fibres in *Arabidopsis* (Blaschek *et al*., 2023) or CoLAC1 for the S2 layer in compression wood tracheids of *Chamaecyparis obtusa* (Hiraide *et al*., 2021). The spatial regulation of lignin concentration is regulated with nanometre precision and has either been conserved, converged or diverged depending on the cell wall layer, cell types, organs and plant species.

Lignin chemistry has also diversified during plant speciation with the evolutionary acquisition of new cell types and morphotypes possessing novel properties and/or providing new functions. Nonconducting xylem fibres provide additional mechanical reinforcement to plant organs and have lignin enriched in units with C_6_ S substitution (Table 1). This chemical attribute has evolved convergently several times during plant speciation and is observed: in *Selaginella* and some species of *Isoetes* but not in other lycophytes (Weng & Chapple, 2010; Rencoret *et al*., 2021); in some fern species, such as *Pleocnemia irregularis* and *Stenochlaena palustris*, but absent from others (Ali *et al*., 2025); in Gnetales (such as *Ephedra fragilis*; Rencoret *et al*., 2021) but not in conifers, cycads and gingko (Terashima *et al*., 2009; Nawawi *et al*., 2017); and in all angiosperms at various levels. Similarly, cells forming the seed coat/testa develop highly lignified secondary walls to reinforce and waterproof the outermost layers of seeds. In different eudicots (e.g. *Cactaceae*, *Cleomaceae* and *Euphorbiaceae*) and monocots (e.g. *Orchidaceae*), seed coat lignins are enriched with units with C_6_ catechol substitutions (C and 5H). Among closely related Brassicales, the seed coat of *Cleome hassleriana* specifically accumulates C C_6_C_3_ units (Tobimatsu *et al*., 2013) whereas *Arabidopsis thaliana* seed coats accumulate flavonoids and C_6_ G phenylpropanoid dimeric units (Yonekura‐Sakakibara *et al*., 2021). This chemical diversity in lignin composition illustrates that seed coat lignification evolved recently, frequently and either divergently or convergently within and between plant lineages. In contrast to these differences in lignin chemistry between plant species, all vascular plants have mechanically reinforced water‐conducting tracheary element cells with lignins similarly enriched in G C_6_C_3_ units (Pesquet *et al*., 2019) conserved from lycophytes to angiosperms. Similarly to lignin concentration, the spatial regulation of its chemistries has either been conserved, converged or diverged depending on the cell wall layer, cell types, organs and plant species.

The tight spatial control of specific lignin chemistries is conceded to depend on both the availability of oxidisable phenolic monomers at a specific time and the spatially restricted oxidative capacity of each cell wall layer (Fig. 2). Distinct combinations and stoichiometries of PRX and/or LAC paralogous proteins with distinct substrate specificities are embedded in each cell wall layer of each cell type to control the local oxidative capacity (Fernández‐Pérez *et al*., 2015; Rojas‐Murcia *et al*., 2020; Hiraide *et al*., 2021; Blaschek *et al*., 2023). In *Arabidopsis*, AtPRX64 and AtLAC12 are associated with primary cell wall layers, whereas AtLAC4 and AtLAC17 are differently distributed between the S1/S2 and S3 secondary cell wall layers of xylem interfascicular fibres (Hoffmann *et al*., 2020; Blaschek *et al*., 2023). In *Arabidopsis* endodermal cells, multiple PRX paralogous isozymes (PRX3, PRX9, PRX39, PRX72 and PRX64; Lee *et al*., 2013; Rojas‐Murcia *et al*., 2020) are responsible for lignin formation in Casparian strips, while the simultaneous loss‐of‐function of nine LAC paralogues had no effect (*LAC1*, *LAC3*, *LAC5*, *LAC7*, *LAC8*, *LAC9*, *LAC12*, *LAC13* and *LAC16*; Rojas‐Murcia *et al*., 2020). Different paralogous proteins moreover display differences in their substrate range preference: AtLAC4 favours coniferyl alcohol compared with AtLAC17 favouring coniferaldehyde in *Arabidopsis* stems (Ménard *et al*., 2022); CoLAC1 preferentially oxidise *p‐*coumaryl alcohol, whereas CoLAC3 prefers coniferyl alcohol in tracheids of *Chamaecyparis obtusa* (Hiraide *et al*., 2021); ChLAC8 favours caffeyl alcohol in the seed coat of *Cleome hassleriana* (Wang *et al*., 2020); and AtLAC5 preferentially enables the incorporation of coniferyl alcohol in *Arabidopsis* seed coats (Yonekura‐Sakakibara *et al*., 2021). The different paralogous LACs and PRXs proteins display both nonredundant localisation and distinct substrate preferences, consequently enabling specific cell wall layers of distinct cell types to accumulate controllable lignin concentrations and chemistries.

Both the number of chemically distinct lignin polymers and their degree of polymerisation (DP) in each cell wall layer and cell type remain unknown. As a bulk, unit chemistry directly affects the molar weight of isolated lignins with high levels of C_6_C_3_ aldehyde units in *CINNAMYL ALCOHOL DEHYDROGENASE (CAD)‐*deficient plants, leading to decreases in molar weight in *Arabidopsis*, poplar, pine and mulberry when compared to the high levels of C_6_C_3_ alcohol units in wild‐type (WT) plants (Dimmel *et al*., 2001; Baumberger *et al*., 2002; Jourdes *et al*., 2007; Madigal *et al*., 2025). Specific tissues/organs such as seed coats of *Cleome hassleriana* contain homopolymers made of only units with C C_6_C_3_ alcohol linked by benzodioxane, together with heteropolymers composed of G and S C_6_C_3_ alcohol units differently linked (Tobimatsu *et al*., 2013). Similarly, aerial roots of vanilla present unusual lignin polymers with an extremely high level of tricin units primarily connected by β‐*O‐*4 linkages (*c*. 96%), whereas the lignin from nodes and internodes showed conventional heteropolymers composed of G and S C_6_C_3_ alcohol units with 3‐ to 5‐fold lower tricin unit levels and only 65–73% of β‐*O‐*4 linkages (Li *et al*., 2022). Importantly, vanilla aerial roots lignin showed a much lower molecular weight than stem lignin, which was mainly explained by the higher incorporation of tricin units providing termination properties to the lignin polymer.

The spatial differences in lignin concentration and chemistry require the immobilisation of lignins to specific cell wall layers. This process has been hypothesised to depend on initiation/nucleation sites that have not yet been properly identified and/or on the anchoring of growing lignin polymers. These options depend on:

1. Specific LCCs varying with the cell wall layer‐specific polysaccharides – including ferulate, *p‐*coumarate and tricin bridges with hemicelluloses (Nishimura *et al*., 2018; Tarasov *et al*., 2018; Kang *et al*., 2019; Mikhael *et al*., 2020).
2. The presence of cell wall‐localised proteins capable of binding specific optically active phenolic di/trimers, called DIRIGENT proteins (DIR) (Hosmani *et al*., 2013; Y.Q. Gao *et al*., 2023).

The initial hypothesis for the intervention of DIRs in lignification as initiation sites to enable the first dimerisation (Davin *et al*., 1997) contradicted the racemic nature of lignins. For DIRs intervention to maintain the lack of optical activity of lignins, similar stoichiometry/affinity of DIR complexes accepting opposite stereoisomers is required. However, some reports also show differences in lignin optical activity (*c*. 15% lower *threo* form proportion) for internal H and G C_6_C_3_ alcohol units in Norway spruce during development (Wadenbäck *et al*., 2004). The loss‐of‐function of DIRs in *Arabidopsis esb1/dir10* and *dir12* mutants resulted in misplacement and dysfunction of the lignified Casparian strips as well as in a reduction of lignified seed coat permeability, thus indicating DIR importance for the lignin‐dependent function of certain cell types (Yonekura‐Sakakibara *et al*., 2021; Y.Q. Gao *et al*., 2023). As lignin formation *in vitro* does not require an initiating primer with a specific structure, we hereby propose another explanation to reconcile DIRs intervention with the racemic nature of lignins. DIRs serve as selective anchoring points embedded in distinct cell wall layers for distinct chemistry (lignin local structure will thus be stereospecific with 50% of dimeric structure fitting for specific DIR structures, either (+) or (−) for β‐β linked dimers for DIR6 in *Arabidopsis*; Gasper *et al*., 2016). Belonging to small multigenic families (26 paralogues in *Arabidopsis*; Paniagua *et al*., 2017) and binding phenolics as trimeric protein structures, combinations of specific DIRs could be genetically controlled to accumulate in different cell wall layers depending on both cell type and environmental conditions. Our hypothesis is supported by the observations that: the factor limiting the activity of DIRs is the release of phenolic dimers (Halls *et al*., 2004); the localisation of DIRs varies between cell wall layers (Davin & Lewis, 2000); and that specific cell wall layers of different cell types have the capacity to bind distinct phenolic dimers (Attoumbré *et al*., 2010). It is, however, unclear whether LCCs and/or DIRs intervene at the initiation and/or anchoring of lignification to control lignin spatial accumulation (Fig. 3b).

To ensure that lignin does not prematurely set the hygroscopic properties of cell walls during the differentiation of each cell type, lignification is tightly temporally regulated to occur after the deposition of cell wall polysaccharides. This process occurs directly in the walls of cells independently of their protoplasts, such as during the *postmortem* lignification of xylem tracheary elements (Pesquet *et al*., 2013) or following concentrically the deposition of primary to secondary cell wall layers in living xylem fibres (Donaldson, 2001) (Fig. 1). This temporal regulation of lignification between cell wall layers is conceded to depend either on a delayed activation of cell wall embedded PRXs/LACs (Blaschek & Pesquet, 2021) and/or on a temporal regulation of the supply of oxidisable lignin precursors (Zhuo *et al*., 2022). Temporal activation of PRXs/LACs has been suggested to depend on proteolytic activation by removing presequences with inhibitory activity, changes in local pH and/or changes in co‐substrate (H_2_O_2_ or O_2_) and/or co‐factor (Fe, Cu and haem) availability (Lee *et al*., 2013; Blaschek & Pesquet, 2021; Pérez‐Antón *et al*., 2022). In parallel, the temporal regulation of the presence of incorporable lignin monomers in each cell wall layer/site of each cell type depends not only on the cell autonomous export of monomers called autocrine, but also on noncell autonomous supply by other ‘good neighbour’ cell types. This cooperative supply can occur either directly surrounding the lignifying cell, called paracrine, or connected through the vascular system, called endocrine (Fig. 2; Pesquet *et al*., 2013; Smith *et al*., 2013; Serk *et al*., 2015). Although monomer supply is key to lignin assembly, mechanisms enabling the selective and controllable transport of chemically distinct monomers during the differentiation of each cell type remain unknown.

**Fig. 5:**
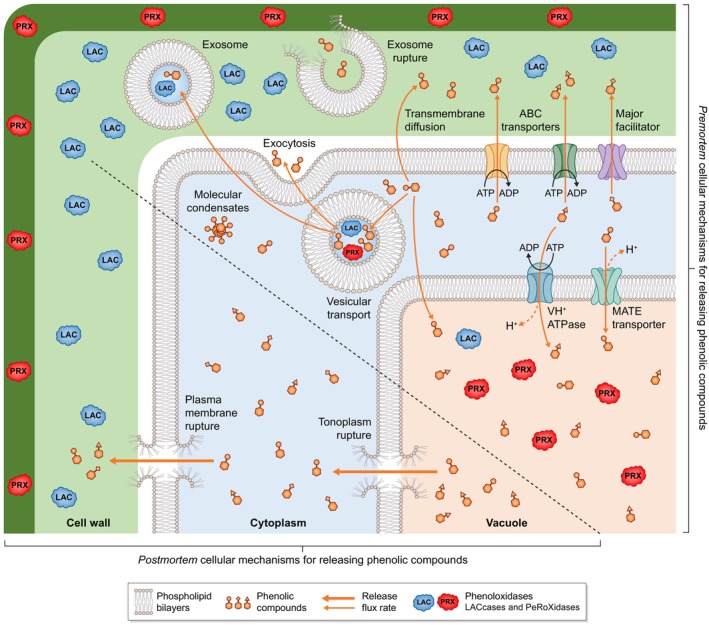
Lignin precursors are transported/released to cell walls for lignification by multiple cellular mechanisms. Schematic representation of cellular mechanisms enabling the transport of phenolic compounds within cells and their release into the cell wall, which is embedded with the phenoloxidases associated with lignification (PRX = peroxidases in red and LAC = laccases in blue). Differences in lignin monomer chemistry are indicated by various shapes associated with the orange hexagons. The direction and relative intensity of the fluxes are indicated by the orientation and the size of the orange arrows between cellular compartments. Phenolic compounds release has first been separated by the cell status either living (*premortem*) or dead after programmed cell death/damage (*postmortem*). The different mechanisms of extracellular release of lignin monomers include *postmortem* high flux leakages during membrane rupture and, for *premortem*, differential fluxes depending on diffusion (due to metabolic gradient caused by the asymmetrical distribution/activity of phenoloxidases), multiple transmembrane transporters (i.e. Vacuolar proton ATPases, ATP binding cassette (ABC) transporters, multidrug and toxic compound extrusion (MATE) and major facilitator transporters) and vesicle transports (exosomes, autophagosome and Golgi‐derived vesicles).

The current biological evidence indicates the existence of multiple transport mechanisms enabling the selective control of lignin monomer concentrations depending on their chemistry. To date, transport mechanisms include (Fig. 5):

1. Passive diffusion across membranes due to metabolic gradients (from cytoplasm to vacuole, vesicles and/or apoplast due to differential distribution and activity of phenoloxidases) specifically for unconjugated phenylpropanoids with alcohol and aldehyde functions as well as for tricin (Vermaas *et al*., 2019; Perkins *et al*., 2022).
2. Chemoselective transmembrane transporters, such as *Arabidopsis* ABC‐G29 for *p‐*coumaryl alcohol (Alejandro *et al*., 2012), *Medicago* ABC‐G10 for *p‐*coumarate (Biała *et al*., 2017), *Larix* ABC‐G36 for coniferyl alcohol (Sun *et al*., 2024), *Cleome* Major Facilitator transporters PLT3 and SUC1 transporting caffeyl alcohol (Zhuo *et al*., 2024), *Phyllostachys* vesicle‐associated V‐ATPase for monolignol 4‐*O‐*glucosides (Tsuyama *et al*., 2019; Shimada *et al*., 2021) or *Picea* vesicle‐associated MATE‐transporters for coniferin (Väisänen *et al*., 2020).
3. Vesicular transport derived from the *trans* Golgi network and/or autophagosomes containing phenolic compounds in various levels of polymerisation from monomers to oligomers (Pickett‐Heaps, 1968; Biała & Jasiński, 2018; Jeon *et al*., 2023; Kankaanpää *et al*., 2024).

Another generally overlooked mechanism of phenolic compound release to cell wall layers is coupled to the programmed cell death of tracheary elements and xylem fibres (Fig. 5; Courtois‐Moreau *et al*., 2009; Ménard *et al*., 2015). Analysing the progression of lignification in differentiating tracheary elements by live cell imaging revealed that lignin accumulation in secondary cell walls just follows programmed cell death within 20 min (Fig. 1; Pesquet *et al*., 2010; Derbyshire *et al*., 2015). Lignification in tracheary elements could be blocked by preventing the normal progression of programmed cell death (Pesquet *et al*., 2013). Note that the release of lignin precursors by cell death can also be due to stress/damage‐induced cell death (i.e. damage by herbivory and cell death due to hypersensitive response). However, to date, the relative contribution of each transport mechanism for the differential lignification of each specific cell wall layer and cell type remains unknown.

## V. Spatial and temporal distribution of lignins with specific topochemistries

**Fig. 6:**
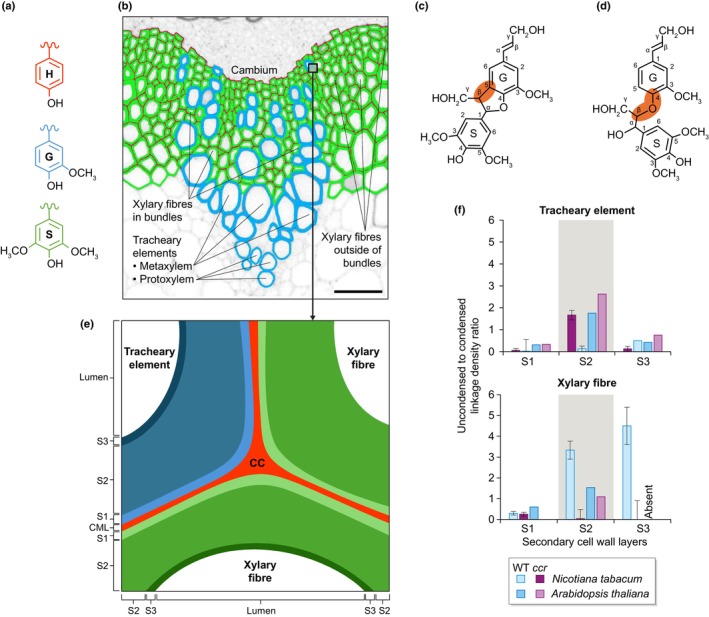
Differences in lignin unit chemistry and linkages between different cell wall layers and cell types are conserved between plant species. (a, b) Schematic representation of lignin spatial distribution. (a) The most abundant lignin units based on their C_6_ substitution include non‐*meta* substituted *p‐*hydroxyphenyl (H) in red, mono‐*meta‐*methoxylated guaiacyl (G) in blue and di‐*meta‐*methoxylated syringyl (S) units in green. (b) Lignins distinct unit enrichment between cell types and cell wall layers are colour‐coded for xylem tissues of *Arabidopsis thaliana* stem cross‐sections. Bar, 100 μm. Different cell types are also indicated, including the different morphotypes of tracheary elements (proto‐ and metaxylem) and fibres within and in‐between vascular bundles (also called fasciculi). The grey box indicates the close‐up presented in (e). (c, d) Model lignin compound varying in linkage type between G and S C_6_C_3_ alcohol units used to produce antibodies. The differences in ‐C‐C‐ condensed β‐5/phenylcoumaran (c) or –C‐O‐C‐ uncondensed β‐*O*‐4/β‐aryl ether (d) linkages between units are shown in orange. (e) Schematic representation of three neighbouring xylem cell types and their different primary (with compound middle lamella (CML) and cell corner (CC)) and secondary cell wall layers (ranging from the most external S1 to the most internal S3), each exhibiting differences in lignin C_6_ unit chemistry with enrichment in H in red, G in blue and S in green. (f) Changes in the relative linkage type ratios between cell wall layers of distinct cell types in wild‐type and *CINNAMOYL‐CoA REDUCTASE* (*CCR*)‐reduced plants using immunodetection with gold‐particle densitometry for antibodies specific to each lignin model compound but with different affinity. Error bars indicate SD. Note that the relative ratios are very conserved between the cell wall layers of each specific cell type for vascular plant species from different families (Brassicaceae *Arabidopsis thaliana* and Solanaceae *Nicotiana tabacum*). Densitometric data taken from Ruel *et al*. (2001, 2009).

The complex topochemical regulation of lignin deposition ensures that different cell types incorporate the distinct lignin chemistry and structure required to diversify their cell wall hygroscopy. In angiosperms, lignin unit C_6_ *meta* substitution levels differ between cell wall layers and cell types. It is generally observed that: units with H rings preferentially accumulate in the primary cell wall/middle lamella in‐between xylem cells; units with G rings are enriched in the secondary cell walls of xylem tracheary elements; and units with S rings are abundant in the secondary cell walls of xylem fibres (Fig. 6a,b; Blaschek *et al*., 2020a). Similarly, units C_3_ aliphatic functions also differ between cell types and cell wall layers, with high aldehyde content in the primary cell wall/middle lamella compared with high alcohol enrichment in secondary cell walls of xylem fibres (Peng & Westermark, 1997; Hänninen *et al*., 2011; Blaschek *et al*., 2020b). The incorporation of these different lignin units also differs during the progression of cell wall maturation (Terashima & Fukushima, 1988; Ménard *et al*., 2022). In xylem tracheary elements, first units with H rings are incorporated in the outermost cell wall layers and gradually progress inwardly, followed by units with G rings and finally S rings; concomitantly, units with terminal aliphatic alcohols are first incorporated and then followed by units with terminal aliphatic aldehydes at later stages (Terashima & Fukushima, 1988; Ménard *et al*., 2022).

Changes in lignin structure between cell wall layers of each cell type also involve different proportions of uncondensed β‐*O‐*4 to condensed (e.g. 5–5, β‐5) linkages (Fig. 6c–f). These differences in lignin condensation vary during the progression of lignification, with highly condensed polymers at the beginning of lignin deposition compared with later stages for secondary cell walls of pine tracheary elements (Terashima & Fukushima, 1988; Fukushima & Terashima, 1991). Immunological quantification using antibodies against G/S C_6_C_3_ alcohol condensed and uncondensed lignin structures revealed a concentric increase in uncondensed linkage proportion from the outermost thin S1 layer to the innermost thin S3 layer in xylem fibres of tobacco and *Arabidopsis* (Fig. 6f; Ruel *et al*., 2001 and Ruel *et al*., 2009). By contrast, tracheary elements in both species exhibited a different profile, with uncondensed linkage maxima in the S2 layer, sandwiched between lower ratioed S1 and S3 layers (Fig. 6f; Ruel *et al*., 2001; Ruel *et al*., 2009). Genetic reduction in *CCR* expression, increasing acid units and decreasing alcohol units, directly decreased the uncondensed to condensed ratio compared with WT plants by several folds in the S2 of both tobacco and *Arabidopsis* fibres, but caused a decrease in the S2 of tobacco tracheary elements while not affecting *Arabidopsis* tracheary elements (Fig. 6f; Ruel *et al*., 2001; Ruel *et al*., 2009). The tight spatial control of specific lignin linkage formation depends on the chemical nature of apoplastic oxidisable lignin monomers (Table 2), their net concentration in specific cell wall layers at a given time, the local pH conditions, the local concentrations of embedded phenoloxidases, and the type(s) of phenoloxidase present (Méchin *et al*., 2007; Matsumoto *et al*., 2020; Kishimoto *et al*., 2022; Tokunaga & Watanabe, 2023). Lignin thus represents a paradox challenging the general protein‐guided law governing other biopolymers – formed by combinatorial assembly at the molecular level but with a controlled compositional and structural accumulation at the subcellular level. This uniquely controlled spatial deposition of lignins with chemically distinct structures provides new properties to the polymer.

## VI. Tuning the hygroscopy of cell walls with lignins to adjust biomechanical properties

**Fig. 7:**
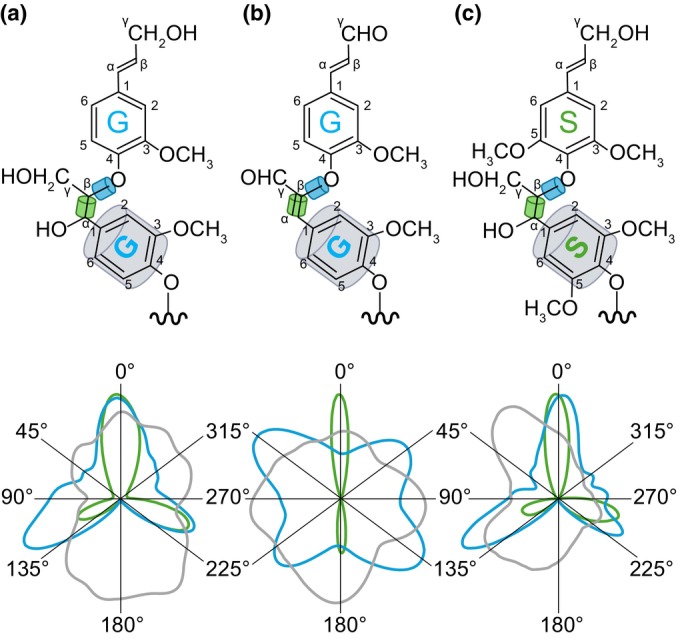
Lignin unit chemistry and interlinkage types adjust the polymer spatial torsion. (a–c) Range normalised distribution changes of torsion angles from 0 to 360° in lignin homoheptamers made of G or S C_6_C_3_ units with terminal aliphatic alcohol or aldehyde joined by uncondensed β‐*O‐*4/β‐aryl ether linkages. The torsion angles shown are between two atoms in the interlinkage including between C_α_ and C_6_ ring in grey, C_α_ and C_β_ in green, and C_β_ and *para*‐*O* of C_6_ ring in blue for homoheptamers of 2 : 4 *erythro*:*threo* of coniferyl alcohol (a), 2 : 4 *cis*:*trans* of coniferaldehyde (b) and 2 : 4 *erythro*:*threo* of sinapyl alcohol (c). Molecular dynamic simulations data taken from Ménard *et al*. (2022).

Lignin deposition will generate novel biophysical properties in each cell wall layer by adjusting cell wall water hygroscopy and cell wall biomechanics. The tuning of these properties will depend on the molecular torsion capacity and the structure of each lignin polymer that varies due to the following:

1. The linkage type between each unit (Table 2; Figs 2, 3, 4, 6, 7; Boerjan *et al*., 2003).
2. The chemical nature of the aliphatic terminal function that always cyclises for alcohols in β‐β and β‐5 linkages, sometimes for acids in both linkages and sometimes for aldehydes, but only in β‐5 linkages (Fig. 4; Holmgren *et al*., 2009; Matsushita *et al*., 2016; Yoshioka *et al*., 2024). Additionally, changes in C_6_ substitution levels, C_3_ terminal function and C_3_ unsaturation directly affect unit bond lengths and, consequently, bond dissociation energy, thus influencing the strength of interunit linkages (Huang *et al*., 2015).
3. The position of each unit in the backbone and/or branches along the polymer sequence shown to differ between units in bulk lignin analyses (such as the preferential presence of β‐*O‐*4 linked H C_6_C_3_ alcohol units at the polymer termini with free C_6_ rings; Kishimoto *et al*., 2010; Mir Derikvand *et al*., 2008; Bouvier d'Yvoire *et al*., 2013; or the preferential presence of β‐*O‐*4 linked G/S C_6_C_3_ aldehyde units at the polymer termini with free C_3_ chains; Sibout *et al*., 2015; Yamamoto *et al*., 2020) and between the different lignified cell types and morphotypes (Yamamoto *et al*., 2020; Pesquet *et al*., 2024). Note that our current knowledge of unit position in a single chain of lignin is still extremely limited.
4. The density and type of molecular interactions (π‐π stacking, H‐ and/or covalent bonds) not only within and between lignins (Notley & Norgren, 2012) but also with cell wall polysaccharides through LCCs (Tarasov *et al*., 2018; Kang *et al*., 2019), such as α‐*O‐*mannose links in conifer wood (Nishimura *et al*., 2018) or ferulate and *p‐*coumarate bridges in monocots (Boerjan *et al*., 2003).

Molecular dynamic simulations showed that lignin β‐*O‐*4‐linked homooligomers of G C_6_C_3_ units have a globular highly compacted structure with terminal alcohol but have a lamellar extended structure with terminal aldehydes (Fig. 7; Ménard *et al*., 2022). Changes in the C_3_ terminal function also affect lignin intramolecular interactions, favoured for terminal alcohols between ‐OH and ‐aryl groups in contrast to terminal aldehydes (Notley & Norgren, 2012). These macromolecular changes due to lignin unit chemistry are directly observable between the cell wall layers. Lignins stained with KMnO_4_ and observed using transmission electron microscopy form punctuate globular structures in primary cell walls but elongated lamellar deposits in secondary cell walls of tracheary elements of *Coleus*, *Pinus*, *Picea* and *Fagus* (Donaldson, 2001). These intra‐ and intermolecular structural changes directly affect the hygroscopic capacity and overall biophysical properties of lignins (Ménard *et al*., 2022; Blaschek *et al*., 2023).

**Fig. 8:**
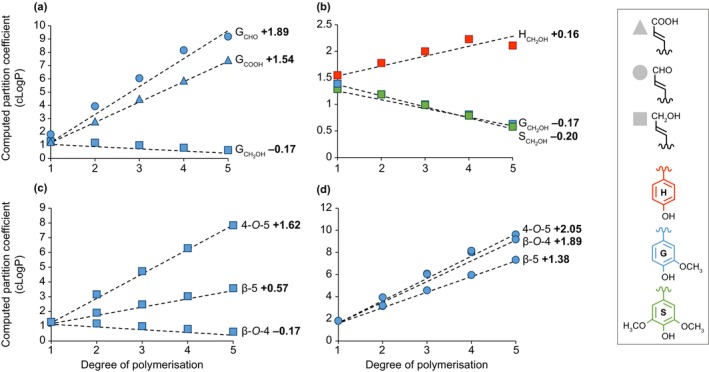
Unit chemistry, linkages and size of lignins adjust polymer hygroscopy. (a–d) Changes in computed octanol/water log partition coefficient (cLogP) using https://www.molinspiration.com/ of lignin oligomers with increasing degree of polymerisation (DP) for specific interlinkage types and unit chemistries. Note that the rates of cLogP changes during the extension of lignin oligomers vary differently depending on unit C_3_ chemistry for G units linked by β‐*O‐*4 linkages (a), on unit C_6_ chemistry for alcohol units linked by β‐*O‐*4 linkages (b), on interlinkage type for G alcohol units (c) compared with G aldehydes (d). The presented modelling data are available in Supporting Information Dataset S1.

**Fig. 9:**
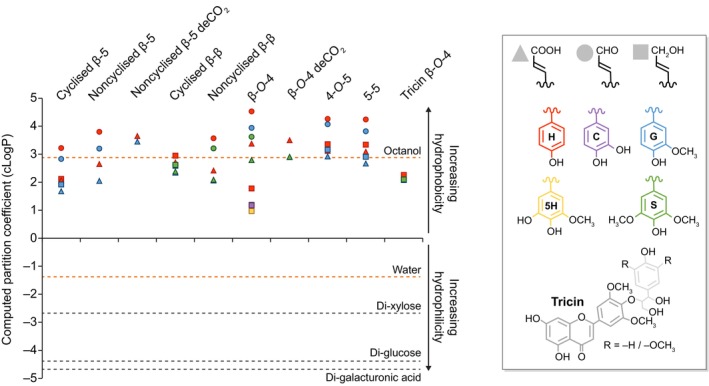
Lignin hygroscopy depends on linkage types and unit chemistry. Computed log partition coefficient (cLogP) of different dimers, differing in unit chemistry and interlinkage type, in octanol/water using https://www.molinspiration.com/. While only representing an approximation, lignin dimers (including dilignols and other dimeric structures) nevertheless exhibited positive values indicating hydrophobicity in contrast to the negative value (indicating hydrophilicity) of dimers of glucose (cellobiose), galacturonic acid (digalacturonan) and xylose (xylobiose) representing cellulose, pectins and hemicelluloses, respectively (indicated with black dotted lines). Additionally, the cLogP of both water and octanol is added as orange dotted lines. Note that the presence of catechol (C and 5H) lignin units limits their interlinkage to β‐*O*‐4/α‐*O*‐5 (Chen *et al*., 2012), whereas tricin linked to C_6_C_3_ alcohol units (R group being either –H or –OCH_3_) is restricted to β‐*O*‐4 (Lan *et al*., 2015; Lui *et al*., 2020; Rencoret *et al*., 2022). Note that C_6_ and C_3_ unit chemistry (shown using colours and symbol, respectively) and interlinkage type changes cLogP values by up to 3.5 units from 0.99 to 4.53. In the case of units with aldehyde and carboxylic acid C_3_ terminal functions, dimeric structure can either cyclise or not (indicated by cyclised or noncyclised) as well as be decarboxylated for acids (indicated by deCO_2_). The presented data are available in Supporting Information Dataset S1.

The water adsorption/retention of cell walls directly depends on lignin unit chemistry. By measuring the contact angle formed by water droplets, spin‐coated synthetic lignin homopolymers made of G C_6_C_3_ units exhibit higher water adsorption/retention with alcohols than with acids, and even lower with aldehydes (Holmgren *et al*., 2009). These differences in wettability are similarly reflected by changes in the calculated partition coefficient (octanol/water logP – with positive values indicating high hydrophobicity and negative values high hydrophilicity) in growing lignin polymers. Homooligo/polymers with G C_6_C_3_ alcohols displayed slightly negative values per added units during extension in contrast to positive values for acids and aldehydes (Fig. 8). The rate of logP changes during the sequential addition of units is affected by both unit chemistry and interlinkage type (Fig. 8). The hygroscopic properties of lignins consequently depend on unit chemistry, linkage between these units and the overall DP. In contrast to the dimeric structure for cell wall polysaccharides, the calculated logPs of dimeric structures of lignins show a much higher hydrophobicity differing with both unit chemistry and with linkage type, cyclisation and decarboxylation (Fig. 9). Changes in unit chemistry also modify lignin mechanical properties with a high stiffness (measured by a high Young modulus) for β‐*O‐*4‐linked homopolymers made of G C_6_C_3_ alcohol units, compared with low stiffness with G C_6_C_3_ aldehyde units (Ménard *et al*., 2022). Density functional theory calculations have shown that linkages between flavonoids and monolignols have similar strength to those between monolignols, with strength depending on the flavonoid substituted groups (Berstis *et al*., 2021). Changes in lignin concentration and chemistry will moreover alter its interaction with other cell wall components, consequently changing the overall cell wall biomechanics (Carmona *et al*., 2015). Lignins will thus contribute to cell wall biomechanics by directly regulating the water imbibition of its polysaccharides and by differentially reinforcing its void spaces depending on their unit chemistries and linkage types.

Lignin spatial distribution between and within cell wall layers is key to controlling their hygroscopic properties. Lowering the lignin concentration of xylem fibres in quintuple loss‐of‐function mutants for *LACs* in *Arabidopsis* increased the water‐dependent swelling of their secondary cell walls (Blaschek *et al*., 2023). Asymmetrical localisation of lignins will differently control the cell and tissue response, such as establishing the molecular brace to guide which parts of the cell wall need to be broken down during the abscission of floral organs (Lee *et al*., 2018; Xue *et al*., 2024). Hinge opening motions during the dehiscence of dry fruits to release seeds also rely on differential lignification. A highly lignified replum (serving hinge pin/knuckle) enables the opening of asymmetrically lignified valves with lignified inner and nonlignified outer margins (serving as hinge leaves). This fruit opening operates with different levels of explosiveness depending on lignin amounts and its spatial distribution: either explosive with an asymmetrical accumulation of lignins in the valve cells in *Cardamine hirsuta* (Pérez‐Antón *et al*., 2022) or nonexplosive with continuous lignification between replum and inner valve margin in *Arabidopsis thaliana* (Liljegren *et al*., 2000). Differences in lignin amounts between cell wall layers and cell types add up to control stem mechanical properties, with high lignin content increasing stiffness but lowering flexibility and breaking point (Ménard *et al*., 2022). Changes in H C_6_C_1_ ester units differently affect poplar trees' response to gravitropic stress, enhancing restoration to upright growth when increased, but lowering the ability to grow straight when diminished (Zhao *et al*., 2021). Enrichment in G C_6_C_3_ units with aldehydes increases stem flexibility in *Arabidopsis* and poplar in contrast to alcohols that increase stiffness (Özparpucu *et al*., 2017, 2018; Ménard *et al*., 2022). By contrast, the decrease in S C_6_C_3_ units with alcohols increases stem stiffness/resistance in the eudicots *Arabidopsis thaliana* and *Brassica napus* but not in the monocot barley (Cao *et al*., 2022; Ménard *et al*., 2022; Shafiei *et al*., 2023). Each plant cell thus has the capacity to specifically control its cell wall lignin content and its lignin chemistry to fine‐tune specific hygroscopic and mechanical properties to fulfil its roles in tissues and organs during plant growth and response to environmental changes.

## VII. Adjusting lignin topochemistry to diversify cell type and tissue functions

The overall lignin concentration poorly predicts general phenotypic traits, like stem height, as they are due to a combination of physiological processes (Blaschek *et al*., 2024). By contrast, specific physiological properties associated with lignins in xylem tissues such as stem lodging (Muszynska *et al*., 2021) and hydraulic vulnerability (Lima *et al*., 2018; Pereira *et al*., 2018; Blaschek *et al*., 2024) are well predicted by the concentration and/or composition of lignins. The modulation of lignin topochemistry greatly impacts many aspects of plant development and physiology. Alteration of proper lignification causes many defects ranging from male sterility (Schilmiller *et al*., 2009; Weng *et al*., 2010), dwarfism (Fig. 4; Zhao *et al*., 2013), seed discoloration (Liang *et al*., 2006; Huang *et al*., 2010), and altered responsiveness to biotic and abiotic stresses (Huang *et al*., 2010; Voelker *et al*., 2010; Ménard *et al*., 2022). The regulation of specific lignin topochemistries is thus an essential part of the differentiation programme of specific cell types and morphotypes acquired by vascular plants but is also fine‐tuned during evolution to enlarge, concentrate, adjust and/or reduce lignin content for specific functions of some species (Boyce *et al*., 2004; Blaschek *et al*., 2024). Lignin units are only partially redundant. For example, H C_6_C_3_ units generally represent a minor portion of lignins and mainly accumulate in response to stresses (Cesarino, 2019). H C_6_C_3_ aldehyde units accumulate in cucumber epidermal cells under pectinase‐mediated elicitation (Varbanova *et al*., 2011) and H C_6_C_3_ alcohol units accumulate in compression wood of conifers submitted to gravitropic stress (Fukushima & Terashima, 1991; Hiraide *et al*., 2021). However, increasing H unit content by different genetic routes in *Arabidopsis* (Vanholme *et al*., 2013; Bonawitz *et al*., 2014; Muro‐Villanueva *et al*., 2022) leads to stunted growth. Increases in 5H units lead to similar stunted developmental defects (Vanholme *et al*., 2010; Weng *et al*., 2010) and restricting unit C_6_ chemistry to C rings in *Arabidopsis* is seedling lethal (Do *et al*., 2007). It is, however, still unclear whether the stunted growth observed is due to the increased accumulation of bioactive phenolic compounds and/or changes in lignin structures/concentrations (Perkins *et al*., 2020). As for any biopolymer, it is not possible to assign unique roles to specific units, as the overall structure and interaction of lignins with other cell wall and cellular components are key to their properties for whole cell functions. Some of the key physiological roles of lignins in plants are listed below:

### 1. Antioxidant and radiation protection

**Fig. 10:**
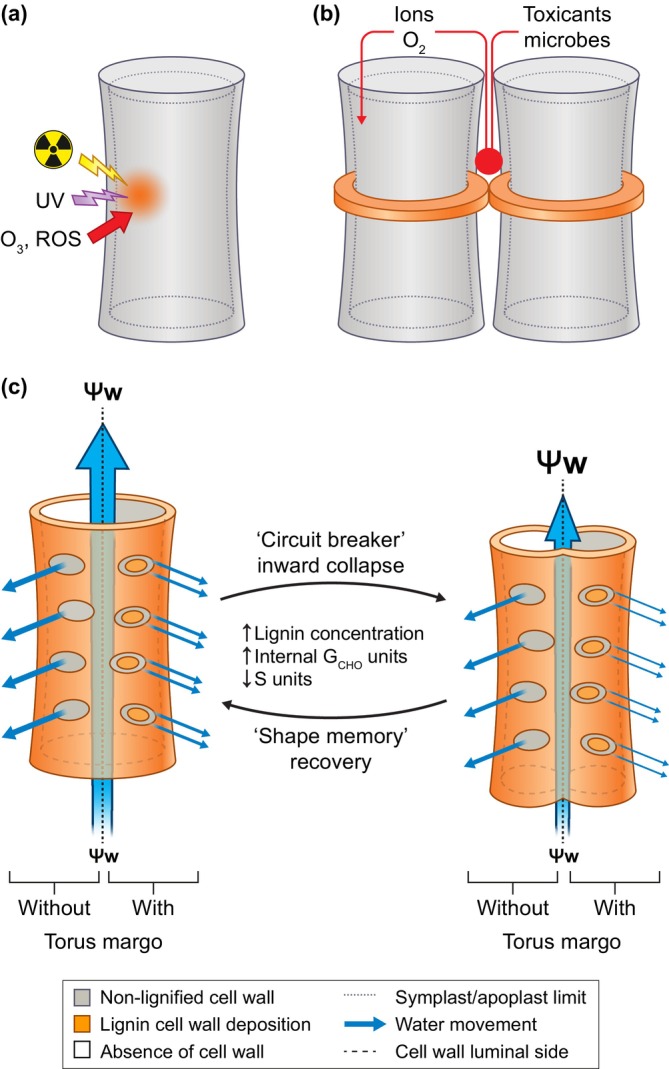
Regulated spatial, structural and compositional formation of lignins enables nonredundant physiological roles for plant adjustment to developmental and/or environmental constraints. Schematic representation in three examples of the physiological role of lignins showing (a) symplast protection due to nonenzymatic/enzymatic cell wall lignification in response to ionising radiations, ultraviolet (UV), ozone and reactive oxygen species (ROS), (b) intercellular sealant to prevent the free diffusion of ions, gases, toxicants and microbes due to lignin deposits such as those observed in endodermis, exodermis, abscission zones and nodules, and (c) tuneable biomechanical responsiveness such as in tracheary element ‘circuit breaker’ to ‘shape memory’ recovery behaviour in response to water availability. Note that the water movement intensity through tracheary element lumens is indicated by the length (indicating the flow rate) and the width of the blue arrows (indicating the amount of water) driven by the water potential (Ψ_w_) gradient. Lateral porosity required for xylem water distribution is shown for both vessel elements (tracheary elements with perforation plates and without torus margo) and tracheids (tracheary element without perforation plates and with torus margo). The impact of lignin unit composition highlights the importance of syringyl (S) and G C_6_C_3_ aldehyde units for controlling tracheary element biomechanical response to Ψ_w_ changes.

This is commonly proposed as the initial function of polyphenolic compounds in living organisms: the protection against denaturating oxidations caused by ionising radiations, UV and/or ROS (Fig. 10a; Labeeuw *et al*., 2015; Renault *et al*., 2017; Renault *et al*., 2019; Sadeghifar & Ragauskas, 2020; Sarosi *et al*., 2021; Tran *et al*., 2021). Lignins represent the ideal antioxidant compounds against external oxidative threats due to their accumulation in the apoplast and their capacity for nonenzymatic oxidation by UV radiations and/or ROS in the presence or absence of ions (Westermark, 1982; Minella *et al*., 2014; Prasse *et al*., 2018). Oxidised lignin polymers will thus be extended with freely available oxidised phenolic compounds and/or cross‐linked with other lignin polymers. Lignins present a low‐energy but broad‐spectrum detoxification mechanism confined in the extra/intercellular space to protect cells/tissues/organs against environmental oxidation. Oxidative stress due to ozone, known to open lignin C_6_ rings when used industrially (Shi *et al*., 2020), increases the condensed to noncondensed linkage ratio of lignin in poplar leaves (Cabané *et al*., 2004; Richet *et al*., 2012). *Arabidopsis pal1pal2* double mutants, strongly reduced in total PHENYLALANINE AMMONIA‐LYASE (PAL) activity and overall lignin content, also show reduced UV tolerance (Huang *et al*., 2010). Presence of heavy metal ions, such as Cu^2+^ or Cd^2+^, directly leads to increased lignin accumulation in roots of *Matricaria chamomilla* (Kováčik & Klejdus, 2008). Upon exposure to excess Fe^2+^/Fe^3+^, which generates ROS (Minella *et al*., 2014), tolerant rice cultivars up‐regulated lignin‐related genes (*PRX*s, *LAC*s and *DIR*) and increased lignification in the outer layers of the cortex and in the vascular bundle when compared to the susceptible cultivars, suggesting that lignin is associated with the tolerance mechanism to excess Fe^2+^/Fe^3+^ (Stein *et al*., 2019). These nonenzymatic mechanisms, such as Ca^2+^‐dependent oxidation of coniferyl alcohol in the presence of superoxide (Westermark, 1982) generated by stresses such as hypoxia (Pucciariello & Perata, 2021), can potentially occur deep within plant tissues. Nonenzymatic oxidation will be limited by the redox potential of phenolic compounds locally present rather than their affinities to phenoloxidases binding pockets, thus offering another level of regulation of lignin topochemistry. In parallel, the ‘in waiting’ enzymatic oxidation using H_2_O_2_‐dependent PRXs embedded in distinct cell wall layers and cell types (Que *et al*., 2018; Blaschek & Pesquet, 2021) also contributes to the antioxidant activity of lignification. It is yet unknown what the overall proportions of nonenzymatically to enzymatically assembled lignins are between cell wall layers and cell types in response to different environmental conditions. Moreover, it remains unclear to which extent nonenzymatic processes contribute to lignin topochemistry and to the hygroscopic and mechanical properties of specific cell types and/or cell wall layers.

### 2. Impermeable sealant

Lignins establish essential sealing barriers surrounding and/or in‐between cells to restrict free movement of ions, toxicants and/or microorganisms across tissues (Fig. 10b). Specifically, lignins are paramount for ensuring the function of the intercellular barriers such as the endodermal Casparian strip (Naseer *et al*., 2012; Lee *et al*., 2013) and the exodermal polar lignin cap (Manzano *et al*., 2025). These specific cell wall reinforcements form tight connections from plasma membrane to plasma membrane between the entire anticlinal circumference of neighbouring cells. Lignified Casparian strips restrictively limit the apoplastic access from the permeable root cortex to its medullar stele. Disturbance of lignin accumulation levels in the Casparian strips causes a loss of apoplastic barrier capacity, enabling free unchecked diffusion into the stele (Naseer *et al*., 2012; Lee *et al*., 2013; Calvo‐Polanco *et al*., 2021). Highly dependent on PRXs rather than LACs (Lee *et al*., 2013; Rojas‐Murcia *et al*., 2020), modifications of lignin accumulation in Casparian strips also lead to the formation of supernumerary compensatory barriers differing in lignin chemistry and displaying distinct ion permeability (Calvo‐Polanco *et al*., 2021). Impermeable barriers are also provided by seed coats, and their reduction in lignin accumulation in *Arabidopsis* by mutating either *LAC5* or *DIR12* greatly compromises their impermeability (Yonekura‐Sakakibara *et al*., 2021). *Arabidopsis* seeds deposit a polar lignin barrier in the outer integument upon cold treatment, which makes the seed coat more impermeable and, thus, enhances seed dormancy by delaying oxidation events that release dormancy (Hyvärinen *et al*., 2025). A similar sealing function of lignins was described for another apoplastic barrier named ‘neck strip’, found in glandular trichomes of different plant species. In cucumber, glandular trichomes produce a white powder known as bloom, which is composed of silica and accumulates at the surface to confer pathogen resistance and to prevent water loss (Samuels *et al*., 1993). The lignin‐based neck strip avoids the apoplastic leakage of silicic acid to stalk and basal cells, allowing its apoplastic accumulation restrictively in the glandular cells. Finally, silicic acid passes through the cuticle and moves to the trichome surface, leading to silica polymerisation and bloom formation (Hao *et al*., 2024). The sealing property of lignins is also associated with dehiscing organs, such as petals, sepals and anthers in *Arabidopsis*, ensuring that the abscission zones will be properly sealed (Lee *et al*., 2018). Lignins have also been hypothesised to limit the access of apoplastic O_2_ to N_2_‐fixing *Frankia* in actinorhizal nodules of *Casuarina glauca*. In contrast to other species, N_2_‐fixing actinorhizal root nodules of *Casuarina* and *Allocasuarina* spp. surround their infected cells with lignins to establish microaerobic conditions required for the function of O_2_‐sensitive nitrogenase (Berg, 1983; Berg & McDowell, 1988; Schubert *et al*., 2013). Following the infection of nodule cortical cells by *Frankia* hyphae, the middle lamella starts lignifying at the penetration site (Berg & McDowell, 1988). Subsequently, the rest of the middle lamella and the primary cell walls of the infected cells lignify, and adjacent uninfected cells deposit a lignified secondary cell wall where in contact with infected cells. PRX activity in the cell walls of both uninfected and infected cells has been detected, but LAC activity has not been examined (Berg, 1983). This specific cell wall lignification creates microaerobic conditions in the infected cells (Berg & McDowell, 1988; Schubert *et al*., 2013), potentially consuming O_2_ to increase lignin concentration using cell wall LACs so that the symplasm remains in suitable microaerobic conditions to enable bacterial N_2_ fixation. It, however, remains unknown how differences in lignin structures due to changes in their chemistry and oxidation ensure liquid and/or gas impermeability in different cells protecting the roots, O_2_‐sensitive nitrogenase in nodules or the abscission zones in plants.

### 3. Tuneable mechanical component

The accumulation of lignins enables the deformations of cell walls independently of (de)hydration levels. This prevents the irreversible agglutination of cell wall polysaccharides during extreme drying (also called hornification) or their extreme swelling into hydrogels when saturated with water (Mo *et al*., 2022). The role of lignins in controlling water‐dependent cell wall deformation of specific cell types is essential for plants to respond to environmental stresses. Specific cell types will alter their lignin chemistry, quantity and distribution to alter the biomechanical properties of their cell wall. During normal growth, xylary fibres require lignins enriched in S C_6_C_3_ units to set their cell wall thickness independently of hydration levels in *Arabidopsis* (Blaschek *et al*., 2023). Under gravitrotropic stress, low to no lignification of fibre cell wall occurs to fully exploit the swelling effect of cell wall polysaccharides, increasing the flexibility but lowering the stiffness of fibres, as observed in gelatinous fibres of hardwood species (Novaes *et al*., 2010). Gymnosperms, devoid of fibres, have normally highly lignified tracheids enriched in G C_6_C_3_ alcohol units. Under gravitropic stress, these tracheids undergo both a change in lignin chemistry and localisation in their compression tissue. Japanese cypress tracheids react by enriching their S2 cell wall layers in H C_6_C_3_ alcohol units (Fukushima & Terashima, 1991; Hiraide *et al*., 2021). This H C_6_C_3_ unit increase in compression wood is also accompanied by more carboxylic acid terminal function and more free phenolic ends (Wei *et al*., 2022).

Other cell types change their lignin chemistry, quantity and distribution to adjust both biomechanical properties and cell wall thickness independently of water availability (Fig. 10c). Modification of tracheary element lignins topochemistries directly adjusts the reversibility of their ‘circuit breaker’ response: setting the levels for inwardly collapsing during drought to close down their lumen and recovering their original luminal area when water availability returns without changing their cell wall thickness both in *Arabidopsis* and red oak (Zhang *et al*., 2016; Ménard *et al*., 2022; Blaschek *et al*., 2023). Systematic analysis of plants across multiple species in many different ecosystems shows that plant hydraulic capacity (reflected by Ψ_50_ corresponding to Ψ_W_ for which 50% of the conductivity is lost) can be improved by −0.3 MPa for every extra percentage of lignins gained in the cell wall of vascular tissues (Pereira *et al*., 2018). Gain in lignin concentration also reduces the susceptibility of vessels to inward collapse (Ménard *et al*., 2022). Plant hydraulic properties also depend on lignin composition, with effects of changing S/G unit content on both Ψ_50_ (reducing Ψ_50_ with S/G increase; Lima *et al*., 2018) and vessel collapse susceptibility (increasing collapse with S/G increase; Ménard *et al*., 2022).

Differences in lignification between cell types will enable unique mechanical properties that have important ecological functions such as the release of seeds during fruit senescence. Due to polarised lignin deposition only in the cell walls of replum and marginal cells compared with the other fruit epidermal tissues, a hinged geometry of lignified cells is formed during *C. hirsuta* fruit ripening to allow a rapid catapult mechanism to release the seeds (Pérez‐Antón *et al*., 2022). Endothecium lignification is also essential to anther dehiscence, by providing structural support for bending and stretching to open the anther (Steiner‐Lange *et al*., 2003; Yang *et al*., 2007). In *Arabidopsis*, this lignification process begins after anther growth and occurs rapidly to enable the timely anther dehiscence and successful pollen release during plant reproduction (Xue *et al*., 2024). These changes in cell wall biomechanics due to lignification are essential for the sessile indeterminate growth and physiology of plants. Once lignified, cells, tissues and organs require minimal energy consumption to mechanically respond to changes in water accessibility/availability.

### 4. Recalcitrance to chemical and enzymatic degradation

An essential property associated with lignins is their resistance to chemical and enzymatic degradation to limit access to cell wall polysaccharides. With fungal cellulases, lignins prevent both the access and the mobility of hydrolytic enzymes along the cellulose microfibrils (Haviland *et al*., 2024). Due to lignin topochemical heterogeneity between cell wall layers and cell types, it currently appears that no specific structural and compositional components of lignins sufficiently accumulate in plants to drive the evolutionary selection of ligno‐degrading organisms (Skyba *et al*., 2013; Cao *et al*., 2022). Lignins thus form the nonsoluble dietary fibres for animal nutrition (Carlsen & Pajari, 2023). Lignins can only be slowly degraded by basidiomycetous white‐rot fungi, mainly via oxidising aliphatic chains and aromatic rings using homologues of the phenoloxidase enzymes that plants use to assemble lignins. Structural analysis between plant lignin assembling LACs and white‐rot fungi disassembling LACs nevertheless reveals major changes in redox potential, substrate binding pocket and access to the active sites (Blaschek & Pesquet, 2021). Changes in lignin concentration affect plant biomass susceptibility to fungal degradation, as natural increases in lignin levels in soybean accessions positively correlate with *Sclerotinia sclerotiorum* stem rot severity (Peltier *et al*., 2009). Changes in lignin chemistry also control its susceptibility to fungal degradation, with enrichment in S C_6_C_3_ units increasing resistance to both brown and white‐rot fungi in poplar overexpressing *FERULATE‐5‐HYDROXYLASE (F5H)* (Skyba *et al*., 2013). By contrast, reducing lignin S/G ratio in rapeseed by knocking down *F5H* leads to high resistance against the stem rot fungus *Sclerotinia sclerotiorum* (Cao *et al*., 2022). Lignins also control bacterial physiology, capable of increasing Gram‐positive *Staphylococcus aureus* sensitivity to β‐lactam antibiotics (Grossman *et al*., 2022) or reducing Gram‐negative *Escherichia coli* cell division rates by stiffening bacterial cell walls (Aguilar‐Sánchez *et al*., 2024). Targeted lignin concentration and S/G increases in kiwi by overexpressing specific *LAC* paralogue similarly reduce plant sensitivity to *Pseudomonas syringae* (Li *et al*., 2025). Genetic engineering of lignin topochemistry directly affects the protective properties of lignins to prevent enzymes from accessing cell wall polysaccharides. Changing G C_6_C_3_ unit aliphatic function from alcohol to aldehyde in ectopically lignified primary cell wall of maize cells reduces their protection of cell wall polysaccharides (Grabber *et al*., 1998). In *Arabidopsis* stems, an inverse linear impact of cell wall polysaccharide protection to hydrolytic enzymes is observed with increasing lignin concentration, S/G proportion, as well as aldehyde to alcohol proportion (Van Acker *et al*., 2013). A general trend for all plants regarding bulk lignin topochemistry cannot, however, be provided to explain their recalcitrance in biomasses due to differences in cell wall layer lignin distribution, lignified cell type proportions, tissue organisation and differences in development. Interestingly, in poplar, a high S/G unit ratio protects against degradation by brown and white‐rot fungi (Skyba *et al*., 2013) but S/G variation in natural poplar variants does not correlate to their catalytic depolymerisation yields (Anderson *et al*., 2019). The combined effects on microorganisms and high recalcitrance to degradation make lignins key elements in the biogeochemical cycle of carbon in our biosphere, determining the decomposition turnover of plant litter into soil and sediments (Huang *et al*., 2023).

## VIII. Conclusions and perspectives

The importance of lignin content and composition/structure between cell wall layers and cell types has been previously undervalued due to averaging effects of bulk analyses. We herein showed that lignin spatial diversity has an essential role in determining the cell wall hygroscopy‐dependent properties of each cell type, allowing plants to thrive in different environments. Previous undervaluation of lignin spatial importance was due to analytical methods that measured lignins on ground samples. Novel quantitative chemical imaging methods with resolution reaching cellular to subcellular levels have been recently developed to characterise lignin chemical diversity *in situ* (Blaschek *et al*., 2024). Specific lignins are tightly regulated in both temporal and spatial manners to set adapted lignin structures to face developmental and/or environmental constraints. These findings are not contradictory to the combinatorial coupling of the extracellular lignin precursor radicals but reveal that lignification represents a paradox depending on its scale; on the one hand, lignin polymers are formed combinatorially through the formation of covalent bonds between two resonance‐stabilised, delocalised radical electrons at the 0.1–1 nm level, but on the other hand, the composition of lignin polymers is tightly controlled spatially at the >100 nm level. Lignins, similarly to other biopolymers, have a chemical code (unit chemistry, unit position and unit linkages) that adjusts their structure and consequently their properties in plant cells. Deeper understanding of the molecular, cellular and tissular mechanisms controlling lignin topochemistry should provide mechanistic comprehension of the lignin code and strategies for using it to select plants with improved biomass to face climate changes without reducing yields.

## Competing interests

None declared.

## Author contributions

Discussions were conducted for several years using physical and online meetings as well as e‐mail exchange between SK, IC, KP and EP. Main conclusions and discussion points were thus included in the present article by EP. All authors then revised and commented on the article.

## Disclaimer

The New Phytologist Foundation remains neutral with regard to jurisdictional claims in maps and in any institutional affiliations.

## Supporting information


**Dataset S1** Modelling values of the effect of degree of polymerisation (DP), linkage type and unit chemistry on the water interaction of lignin di/oligomers using calculated water/octanol log partition coefficients (cLogP).
Please note: Wiley is not responsible for the content or functionality of any Supporting Information supplied by the authors. Any queries (other than missing material) should be directed to the *New Phytologist* Central Office.

## Data Availability

Modelling data presented in Figs 8 and 9 are in Dataset S1.
